# Proteomic Landscape of Tissue-Specific Cyclin E Functions in Vivo

**DOI:** 10.1371/journal.pgen.1006429

**Published:** 2016-11-09

**Authors:** Junko Odajima, Siddharth Saini, Piotr Jung, Yasmine Ndassa-Colday, Scott Ficaro, Yan Geng, Eugenio Marco, Wojciech Michowski, Yaoyu E. Wang, James A. DeCaprio, Larisa Litovchick, Jarrod Marto, Piotr Sicinski

**Affiliations:** 1 Department of Cancer Biology, Dana-Farber Cancer Institute, Boston, Massachusetts, United States of America; 2 Department of Genetics, Harvard Medical School, Boston, Massachusetts, United States of America; 3 Department of Internal Medicine and Massey Cancer Center, Virginia Commonwealth University, Richmond, Virginia, United States of America; 4 Department of Biochemistry and Molecular Pharmacology, Harvard Medical School, Boston, Massachusetts, United States of America; 5 Department of Biostatistics and Computational Biology, Dana-Farber Cancer Institute and Harvard T.H. Chan School of Public Health, Boston, Massachusetts, United States of America; 6 Center for Cancer Computational Biology, Dana-Farber Cancer Institute, Boston, Massachusetts, United States of America; 7 Department of Medical Oncology, Dana-Farber Cancer Institute, and Department of Medicine, Harvard Medical School, Boston, Massachusetts, United States of America; Fred Hutchinson Cancer Research Center, UNITED STATES

## Abstract

E-type cyclins (cyclins E1 and E2) are components of the cell cycle machinery that has been conserved from yeast to humans. The major function of E-type cyclins is to drive cell division. It is unknown whether in addition to their ‘core’ cell cycle functions, E-type cyclins also perform unique tissue-specific roles. Here, we applied high-throughput mass spectrometric analyses of mouse organs to define the repertoire of cyclin E protein partners *in vivo*. We found that cyclin E interacts with distinct sets of proteins in different compartments. These cyclin E interactors are highly enriched for phosphorylation targets of cyclin E and its catalytic partner, the cyclin-dependent kinase 2 (Cdk2). Among cyclin E interactors we identified several novel tissue-specific substrates of cyclin E-Cdk2 kinase. In proliferating compartments, cyclin E-Cdk2 phosphorylates Lin proteins within the DREAM complex. In the testes, cyclin E-Cdk2 phosphorylates Mybl1 and Dmrtc2, two meiotic transcription factors that represent key regulators of spermatogenesis. In embryonic and adult brains cyclin E interacts with proteins involved in neurogenesis, while in adult brains also with proteins regulating microtubule-based processes and microtubule cytoskeleton. We also used quantitative proteomics to demonstrate re-wiring of the cyclin E interactome upon ablation of Cdk2. This approach can be used to study how protein interactome changes during development or in any pathological state such as aging or cancer.

## Author Summary

The proliferation of mammalian cells is driven by proteins called cyclins, which bind and activate their catalytic partners, the cyclin-dependent kinases (Cdks). Cyclin-Cdk complexes drive cell proliferation by phosphorylating several cellular proteins. This study focuses on the E-type cyclins (cyclins E1 and E2). These proteins were shown to play important roles in cell cycle progression, and are often overexpressed in human cancers. It remained unclear, however, whether in addition to their well-established cell cycle roles E-type cyclins perform distinct tissue-specific functions *in vivo*, in the living mouse. To address this question we developed a system, which combines gene targeting in embryonic stem cells with high-throughput mass spectrometry. This approach allowed us to determine the identity of cyclin E protein partners, as well as phosphorylation substrates of cyclin E and its associated kinase, Cdk2, in different mouse organs. These analyses identified several novel, previously unanticipated interactors and phosphorylation substrates of cyclin E-Cdk2, and revealed that cyclin E plays distinct molecular functions in different compartments *in vivo*. This approach can be used to study the *in vivo* function of essentially any protein, in any model organism, and in any physiological and pathological state.

## Introduction

E-type cyclins (cyclins E1 and E2, collectively referred to as ‘cyclin E’) represent components of the core cell cycle machinery. The two E-cyclins are encoded by separate genes, but they show substantial amino acid sequence similarity. In proliferating cells, E-cyclins become upregulated during the late G1 phase. Once induced, E-cyclins bind and activate their catalytic partner, the cyclin-dependent kinase 2 (Cdk2). Cyclin E-Cdk2 complexes phosphorylate proteins involved in cell cycle progression (the retinoblastoma protein pRB, p107, p130, p27^Kip1^), centrosome duplication (NPM1, CP110), histone biosynthesis (p220^NPAT^) and DNA replication (Cdc6, MCMs), thereby driving cell proliferation [1,2]. Consistent with growth-promoting roles for E-cyclins, amplification of the *cyclin E1* and/or *E2* genes and pathological overexpression of cyclin E proteins were documented in a wide range of human cancer types [1].

While the E-type cyclins have been extensively studied using biochemical approaches and in *in vitro* cultured cells, much less is known about the molecular functions of these proteins in different cell types within a living organism. In particular, it is not known whether cyclin E plays distinct molecular functions in different compartments or at different stages of development. Analyses of mice lacking E-cyclins revealed that both cyclin E1-null and E2-null mice are viable and develop relatively normally [3,4]. The only phenotype observed in cyclin E2-deficient mice was a defect in spermatogenesis leading to decreased male fertility. This phenotype was further exacerbated in mice with reduced dosage of cyclin E1 (*E1*^*+/-*^*E2*^*-/-*^), and was most pronounced upon conditional ablation of both E-cyclins in the male germline (*E1*^*Δ*^*/*^*Δ*^*E2*^*-/-*^), indicating that the two E-cyclins perform redundant functions in spermatogenesis [3,5]. Strikingly, the testicular phenotype of cyclin E-deficient mice closely mimics abnormalities seen in knockout mice devoid of cyclin E catalytic partner, Cdk2 [6,7]. These observations strongly suggest that cyclin E-Cdk2 kinase plays an important function during the spermatogenic process. However, the molecular role of cyclin E-Cdk2 in the male germline is largely unknown.

Whereas genetic ablation of individual cyclins yielded viable mice, a combined ubiquitous deletion of both E-type cyclins resulted in an early embryonic lethality [3,4]. This has been taken as an indication that the two E-cyclins perform overlapping functions in normal development. However, the lethality of cyclin E-deficient animals hampered analyses of cyclin E function at later stages of development and in adult organs.

To overcome these limitations, and to investigate the molecular functions of cyclin E in different compartments of the living organism, we developed a system that involves high-throughput proteomic analyses of organs derived from genetically engineered mice. Using this system, we provide insights into tissue-specific molecular roles for cyclin E and its associated kinase, Cdk2 *in vivo*.

## Results

### Generation of tagged cyclin E1 knock-in mice and purification of cyclin E1-containing protein complexes from different mouse organs

In order to elucidate the *in vivo* functions of cyclin E, we decided to generate knock-in mouse strains expressing tandemly (Flag- and hemagglutinin, HA-) tagged versions of cyclin E1 in place of wild-type cyclin E1. We reasoned that these mice would allow us to use tandem immunoaffinity purifications with anti-Flag and -HA antibodies, followed by repeated rounds of high-throughput mass spectrometry, to determine the repertoire of cyclin E1-associated proteins in essentially any tissue or cell type, and at any stage of development.

**Fig 1:**
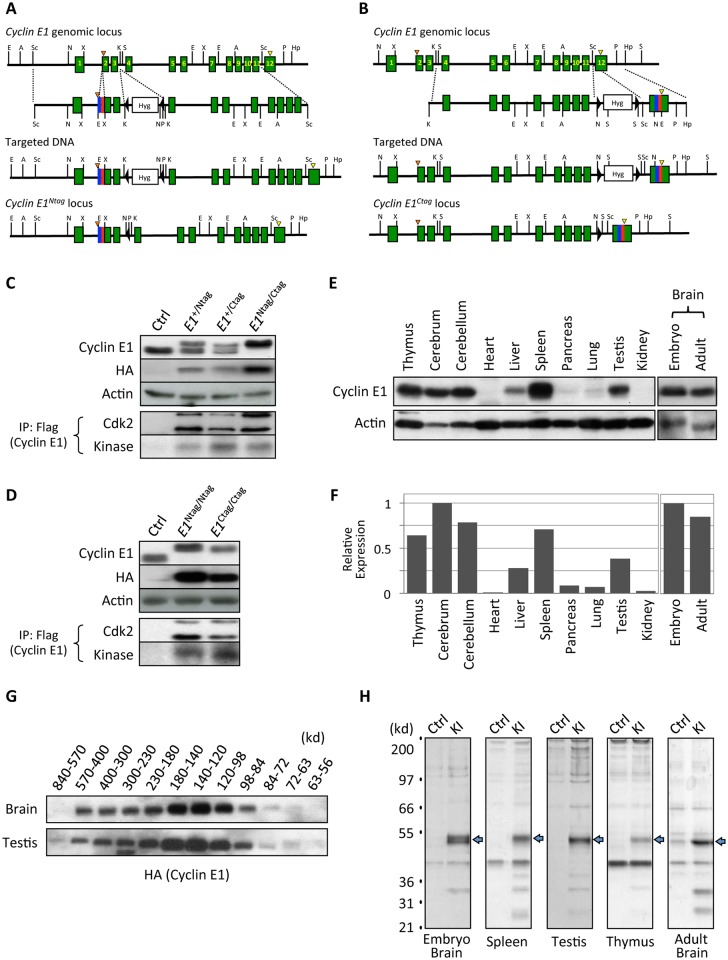
Generation of tagged cyclin E1 knock-In mice and analyses of cyclin E1-containing protein complexes. (A and B) Targeting strategy to knock-in Flag and HA tags into *the cyclin E1* locus to generate N-terminally tagged *cyclin E1*^*Ntag*^ (A) and C-terminally tagged *cyclin E1*^*Ctag*^ alleles (B). The exons are shown as green boxes, Flag tag as a blue box, and HA tag as a red box. Start and stop codons are marked with orange and yellow arrowheads, respectively. The hygromycin resistance cassette (Hyg) with flanking loxP sequences (filled arrows) is also indicated. Restriction enzyme recognition sites: E, EcoRI; A, AflII; Sc, ScaI; N, NotI; X, XhoI; K, KpnI; S, SpeI; P, PmeI; Hp, HpaI. Note that panel (A) was shown in ref [8]. (C) Western blot analysis of wild-type control (Ctrl), heterozygous cyclin *E1*^*+/Ntag*^, *cyclin E1*^*+/Ctag*^, and *cyclin E1*^*Ntag/Ctag*^ embryonic stem cells probed with anti-cyclin E1 and -HA antibodies. Actin served as a loading control. Forth panel: cyclin E1 was immunoprecipitated with anti-Flag antibody and the immunoblots were probed with anti-Cdk2 antibody. Fifth panel: anti-Flag immunoprecipitates were used for *in vitro* kinase reactions using histone H1 as a substrate. (D) Same analyses as in (C) using spleens of homozygous knock-in mice. Lanes 1–2 in panels (C and D) were previously shown in [8]. (E) Cyclin E levels detected by western blotting in the indicated organs of 1-month-old mice and in embryonic brain (day E14.5). Actin served as a loading control. The last two lanes (Brain) were previously shown in [8]. (F) Quantification of cyclin E levels in different organs, normalized against actin (from E). (G) Protein lysates from brains and testes of adult tagged cyclin E1 knock-in mice were separated by gel-filtration chromatography. Fractions containing protein complexes of the indicated molecular weights were analyzed by western blotting for cyclin E using an anti-HA antibody. (H) Cyclin E1-associated proteins were purified from the indicated organs of tagged cyclin E1 knock-in (KI) mice, or from control mice (Ctrl, ‘mock’ purifications) by sequential immunoaffinity purifications with anti-Flag and -HA antibodies, and 10% of the final eluate was resolved on PAGE gels and silver-stained. Arrows indicate bands corresponding to cyclin E1. Panels representing embryonic and adult brains were previously shown in [8].

We inserted DNA sequences encoding Flag and HA tags into the amino terminus of cyclin E1, immediately downstream of the start codon, using gene-targeting in embryonic stem (ES) cells (Fig 1A). Subsequently, homozygous *cyclin E1*^*Ntag/Ntag*^ mice were generated using standard procedures. Since a tag at a particular end of cyclin E1 molecules might destabilize the protein, or render it non-functional *in vivo*, we also generated the second knock-in strain, in which we inserted DNA sequences encoding these two tags into cyclin E1 carboxy terminus, immediately upstream of the stop codon, yielding *cyclin E1*^*Ctag/Ctag*^ animals (Fig 1B). We verified that in the tissues of knock-in mice the tagged cyclin E1 alleles were expressed at the same levels as wild-type cyclin E1 in the corresponding tissues of control animals (Fig 1C and 1D). We also verified that both amino- and carboxy-terminally tagged cyclin E1 retained the ability to bind and to activate cyclin E catalytic partner, Cdk2 (Fig 1C and 1D). Like wild-type cyclin E1, tagged cyclin E1 was expressed at high levels in several organs of adult mice, as well as in embryonic brains (Fig 1E and 1F).

In our proteomic analyses we decided to focus on five compartments: embryonic brains, adult brains, spleens, thymuses and testes, as these organs expressed particularly high levels of cyclin E1 (Fig 1E and 1F and S1 Fig). First, we analyzed protein lysates from mouse organs using size exclusion chromatography, to determine the molecular weight of cyclin E1-containing complexes. We found that cyclin E1 was present in a wide range of protein fractions (56–840 kD), suggesting that it forms a multitude of distinct protein complexes *in vivo* (Fig 1G). We next purified cyclin E1-containing protein complexes from each of the five organs using sequential immunoaffinity purifications with anti-Flag and -HA antibodies (Fig 1H), and identified cyclin E1-associated proteins using repeated rounds of liquid chromatography–tandem mass spectrometry (6–10 independent purifications/mass spectrometry runs). In parallel, we performed the same number of purifications/mass spectrometry analyses from control wild-type animals, which do not express tagged cyclin E1, and the identified proteins were subtracted as a background (see S1 Appendix).

### Studies of cyclin E1-interactomes from different organs

**Fig 2:**
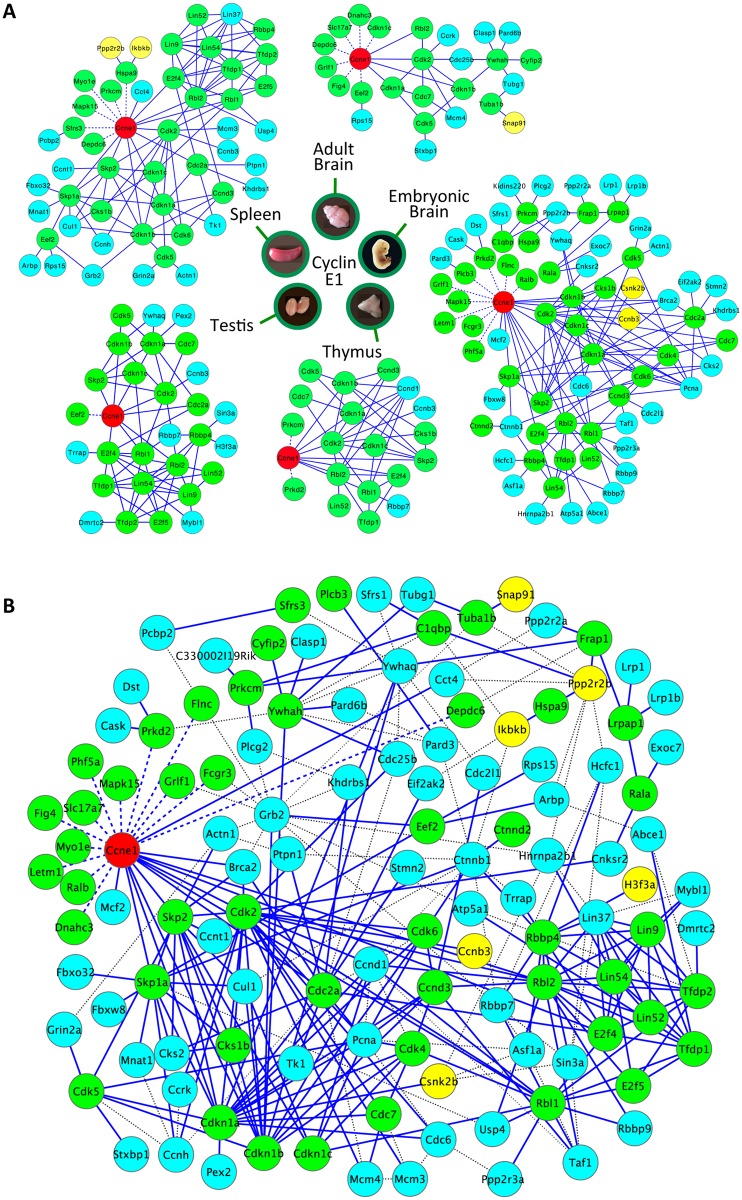
Cyclin E1-interactomes. (A) Diagrams depicting cyclin E1-interacting proteins in the indicated mouse organs. Cyclin E1 is shown as a red node. Green nodes denote highest-confidence ‘core’ interactors (Category 1, see S1 Appendix). Yellow and blue nodes represent, respectively, lower confidence Categories 2 and 3 interactors that were included to the interactome based on their reported interaction with core interactors in the STRING database (see S1 Appendix). Solid lines depict STRING-verified interactions. Dashed lines depict an interaction derived from our mass spectrometry analyses between cyclin E1 and a protein that has no known interactions with other core interactors. (B) A combined diagram depicting cyclin E1-interacting proteins from all five organs analyzed. Cyclin E1 is shown as a red node. Green nodes denote highest-confidence core (Category 1) interactors. Yellow and blue nodes denote, respectively, Categories 2 and 3 interactors, which were included into the interactome based on their ability to interact with core interactors as revealed by STRING (see S1 Appendix). Solid blue lines depict STRING-verified interactions between pairs of proteins that were identified by us as cyclin E1-interacting proteins within the same organ. Gray dotted lines depict STRING-verified interactions between pairs of proteins identified as cyclin E1-interators in different organs. Blue dashed lines depict interactions detected in our mass spectrometry analyses between cyclin E1 and a protein that has no known interactions with other core proteins within the same organ interactome.

**Fig 3:**
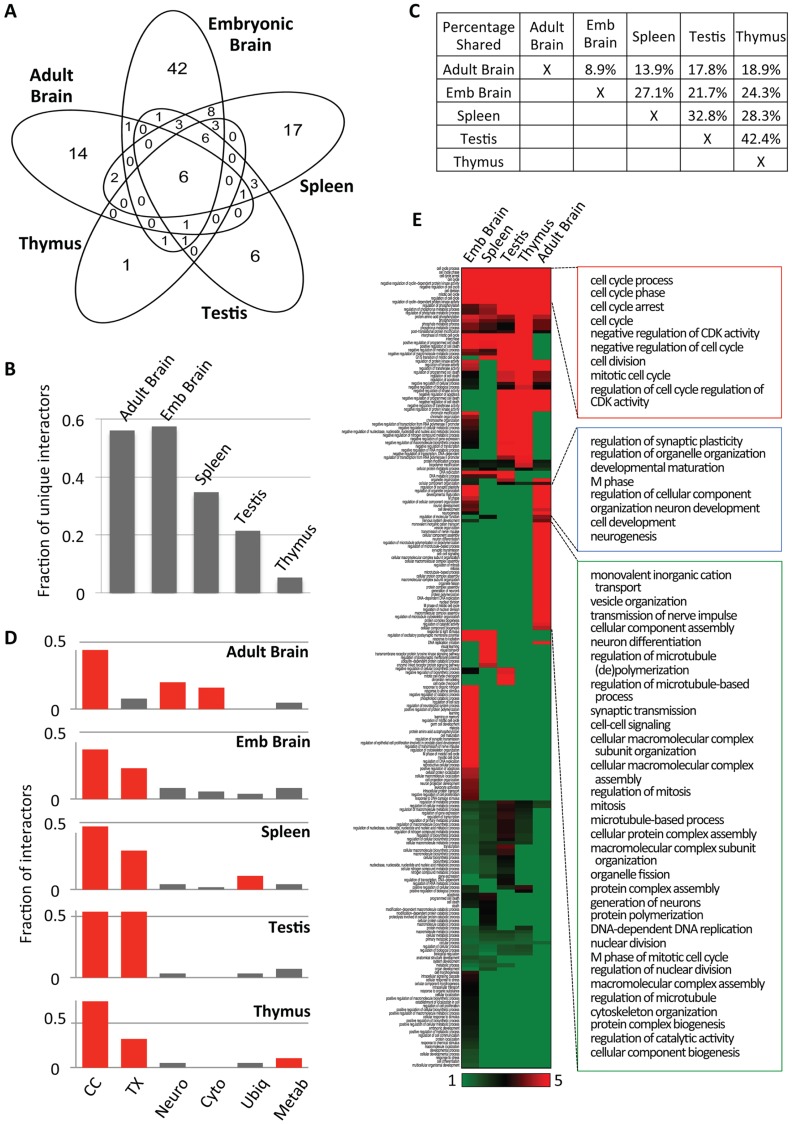
Analyses of cyclin E1-interactomes. (A) Venn diagram depicting the numbers of unique and shared cyclin E1 interactors in the indicated organs. (B) Fraction of unique interactors in the indicated organs. (C) Pairwise comparisons of the fraction of cyclin E1-interactors shared between the indicated organs. (D) The fraction of cyclin E1 interactors in the indicated organs that were assigned to a given Gene Ontology category (see S2 Table). Categories assigned at least 10% of the interactors in a given organ are marked in red. CC, cell cycle; TX, transcription; Neuro, neuronal function; Cyto, microtubules/cytoskeleton; Ubiq, ubiquitination; Metab, metabolism. (E) Heatmap displaying functional enrichment of cyclin E1 interactors in Gene Ontology classes of biological processes. The five columns correspond to the five organs analyzed, and each horizontal row denotes a distinct biological process. Colors depict fold-enrichment for the cyclin E1 interactors from the particular organ in a given biological process, between green (fold-enrichment one or lower) to red (fold-enrichment five or higher). Only categories in which at least one organ had an EASE score of 0.2 or lower are shown (see S1 Appendix). Left panel shows a complete heatmap, right panels show selected common and organ-specific biological processes: cell cycle (red box, enriched in all organs), neurogenesis and synaptic plasticity (blue box, shared between embryonic and adult brains) and regulation of microtubule-based processes and microtubule cytoskeleton (green box, specific to adult brain).

These procedures allowed us to determine the identity of cyclin E1-associated proteins (‘E1-interactomes’) in embryonic brains, adult brains, spleens, thymuses and testes. In total, we detected 117 high-confidence cyclin E1 interactors (Fig 2, S2 Fig and S1 Table). Thirty-seven of these were detected in at least two different compartments (Fig 3A and S1 Table). These shared interactors contained essentially all well-established cyclin E-binding proteins, including Cdk2, ‘pocket proteins’ p107 and p130, as well as cell cycle inhibitors p27^Kip1^, p57^Kip2^ and p21^Cip1^, and were highly enriched for cell cycle proteins (Fig 2 and S1 Table).

In addition to these shared interactors, 80 proteins were found to associate with cyclin E1 only in one organ (Fig 3A). Cyclin E1-interactomes in embryonic and adult brains contained the highest fraction of unique interactors (57.5% and 56.0%, respectively), followed by spleens (34.7%), testes (21.4%) and thymuses (5.3%) (Fig 3B). Surprisingly, the fraction of shared proteins between embryonic and adult brain interactomes was very low (8.9%), suggesting distinct molecular roles for cyclin E in embryonic versus in adult brains. In contrast, testes shared nearly half of their interactors (42.4%) with thymuses and 32.8% with spleens, while the overlap between thymic and splenic interactomes was 28.3% (Fig 3C).

We next assigned Gene Ontology functions to interactors found in different organs. In spleens, thymuses and embryonic brains the most frequent function was cell cycle, while transcription represented the second most frequent category. In addition, over 10% of cyclin E1 interactors in spleens and in thymuses belonged to proteins involved in protein ubiquitination and in metabolism, respectively (Fig 3D and S2 Table). In the testicular interactome, transcription and cell cycle constituted two equally most frequent functions. In the adult brains, in addition to cell cycle proteins, a significant fraction of cyclin E1-interactors represented proteins involved in neuronal function (20%), and 16% belonged to proteins that play roles in regulation of microtubules and cytoskeleton (Fig 3D and S2 Table).

We constructed a biological process enrichment heat map of cyclin E1 interactors (Fig 3E and S3 Table). As expected, cell cycle control category was enriched across all organs. In addition, we observed that apoptosis/cell death and chromatin modification categories were enriched in multiple compartments. Indeed, cyclin E was postulated to play roles in both processes [9–11]. Strikingly, embryonic brain and adult brain interactors shared functions involved in neuron development, neurogenesis and regulation of synaptic plasticity, while only interactors from adult brains showed enrichment for regulation of microtubule-based processes and microtubule cytoskeleton (Fig 3E). These findings indicate that cyclin E plays previously unanticipated functions in neuronal differentiation as well as in regulating microtubules and cytoskeleton in terminally differentiated neurons.

### Cyclin E associates with Cdk1, Cdk2, Cdk5, and Cdc7 in several organs

Cyclin E was previously shown to interact with Cdk2 and, to a lesser extent, with Cdk1 [12]. Indeed, we detected Cdk2 bound to cyclin E1 in all organs, whereas we observed Cdk1 in some (embryonic brains, spleens, testes) but not in other compartments (thymuses, adult brains) (Fig 2A and S1 Table).

Unexpectedly, we observed association of cyclin E with Cdk5 in all five organs studied (Fig 2A and S1 Table). Cdk5, together with its regulatory partners p35 and p39 represents neuronal-specific kinase that plays key roles in neuronal differentiation [13]. Cyclin E1 was shown to interact with Cdk5 in terminally differentiated neurons, where it negatively regulates phosphorylation of synaptic proteins [8]. Our unexpected finding that cyclin E1 interacts with Cdk5 in all compartments studied here indicates that cyclin E-Cdk5 complexes likely play much wider physiological roles than previously anticipated, by acting outside the nervous system.

We also observed, and confirmed by immunoprecipitation (IP)–western blotting, an unexpected association of cyclin E1 with another cell cycle kinase, Cdc7 (Fig 2A, S2A Fig and S1 Table). This protein is known to interact with its regulatory subunits Dbf4 and Drf1, and to play a rate-limiting role in firing mammalian DNA replication origins [14,15]. Importantly, we did not detect Dbf4 or Drf1 as cyclin E1-interacting proteins, suggesting that cyclin E1 forms distinct complexes with Cdc7. Of note, cyclin E-Cdk2 and Dbf4-Cdc7 complexes were postulated to play distinct molecular functions during firing of mammalian DNA replication origins [16]. Our findings raise a possibility that cyclin E may contribute to this process through cyclin E-Cdk2 as well as cyclin E-Cdc7 complexes.

Collectively, these results revealed that cyclin E interacts *in vivo* with a much wider range of cell cycle-related kinases than previously appreciated.

### Re-wiring of the cyclin E1 interactome upon ablation of Cdk2

We reasoned that application of quantitative proteomic methods would allow us to visualize how the cyclin E1 interaction network is perturbed in different pathological states. As a proof of principle, we decided to analyze the changes in cyclin E1 interactome upon ablation of Cdk2, the major catalytic partner of cyclin E. To address this, we bred cyclin *E1*^*Ntag/Ntag*^ mice with *Cdk2*^*+/-*^ animals and generated *Cdk2*^*-/-*^*/cyclin E1*^*Ntag/Ntag*^ mice. We then used spleens and thymuses from *Cdk2*^*-/-*^*/cyclin E1*^*Ntag/Ntag*^ and from *cyclin E1*^*Ntag/Ntag*^ mice (control, *Cdk2*^*+/+*^) to isolate cyclin E1-associated proteins using sequential immunoaffinity purifications, as above. We then compared the abundance of cyclin E1 interactors between these two genotypes using isobaric tags for relative and absolute quantification (iTRAQ) approach. In this method, protein purification products from different samples are labeled with isobaric tags containing different reporter isotopes. After labeling, the samples are mixed and subjected to quantitative mass spectrometry analysis. Analogous peptides derived from each sample are distinguished due to the mass differences of the isotope reporter ions, and the ratio of the reporter ion peak intensities reflects the relative abundance of the peptides in each sample [17].

**Fig 4:**
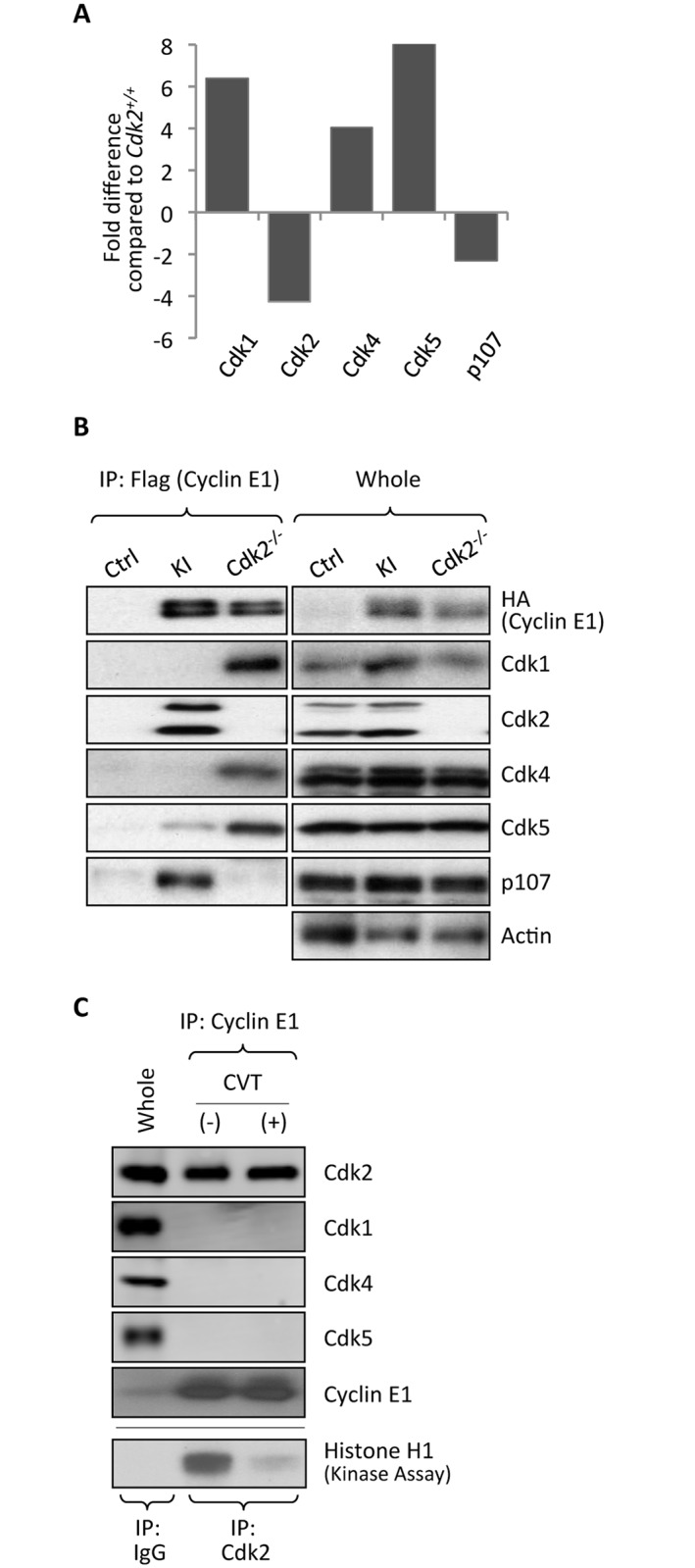
Quantitative proteomic (iTRAQ) analysis of cyclin E1-interacting proteins in mouse organs in the absence of Cdk2. (A) Relative abundance of cyclin E1-associated Cdk1, Cdk2, Cdk4, Cdk5 and p107 in the spleens of *Cdk2*^*-/-*^*/cyclin E1*^*Ntag/Ntag*^ mice, as compared to *Cdk2*^*+/+*^*/cyclin E1*^*Ntag/Ntag*^ animals, was determined by iTRAQ labeling and LC-MS. (B) The amount of cyclin E1-associated Cdk1, Cdk2, Cdk4, Cdk5 and p107 in the spleens of wild-type (Ctrl), *Cdk2*^*+/+*^*/cyclin E1*^*Ntag/Ntag*^ (KI), and *Cdk2*^*-/-*^*/cyclin E1*^*Ntag/Ntag*^ (Cdk2^*-/-*^) mice was gauged by immunoprecipitation with an anti-Flag antibody and immunoblotting with the indicated antibodies. Abundance of each protein in total lysates (whole) is also shown. (C) Spleens from wild-type mice were incubated with 20 μM CVT-313 (+) or with vehicle only (-). Association of cyclin E1 with Cdk2, Cdk1, Cdk4 and Cdk5 was assessed by IP–western blotting. Whole, whole cell lysate from vehicle only-treated mice. Lower panel: To ensure that CVT-313 treatment inhibited Cdk2 kinase activity, Cdk2 was immunoprecipitated from lysates and used for *in vitro* kinase reactions with histone H1 as a substrate. Note that CVT-313 treatment strongly decreased Cdk2 kinase activity.

We found that ablation of Cdk2 led to a strong increase in association of cyclin E1 with several other cyclin-dependent kinases, such as Cdk1, Cdk4 and Cdk5 (Fig 4A and S4 Table). While association of cyclin E1 with Cdk1 in Cdk2-null cells has been reported before [12], increased binding to Cdk4 and Cdk5 was unexpected. We verified using IP–western blotting that in the wild-type setting, cyclin E1 bound predominantly to Cdk2. However, ablation of Cdk2 led to a dramatic increase of cyclin E1-Cdk1, E1-Cdk4 and E1-Cdk5 interactions, both in Cdk2-null spleens and thymuses (Fig 4B and S3A Fig). Importantly, binding of Cdk1 and Cdk4 to their physiological cyclin partners (cyclins B1 and D1, respectively) remained unchanged in Cdk2^-/-^ organs (S3B Fig), arguing against re-distribution of Cdk1 and Cdk4 away from its normal partners to cyclin E, and suggesting that the “free” pool of Cdks bound to cyclin E in the absence of Cdk2. Interestingly, binding of cyclin A2 to Cdk1 was increased in Cdk2^-/-^ cells (S3B Fig). Like cyclin E, cyclin A2 normally interacts with both Cdk2 and Cdk1, and ablation of Cdk2 increases cyclin A2 binding to the Cdk1 subunit.

We also tested the effect of chemical inhibition of Cdk2 kinase activity on the composition of cyclin E-containing complexes. To do so, we used CVT-313, a reversible ATP-competitive inhibitor of Cdk2 (IC_50_ = 0.5 μM), which can also inhibit cyclin Cdk1 with lower potency (IC_50_ = 4.2 μM) [18]. In contrast to genetic Cdk2 ablation, inhibition of Cdk2 activity did not increase the interaction of cyclin E with other Cdks (Fig 4C), indicating that the loss of Cdk2 protein, rather than inhibition of Cdk2 kinase triggered the observed re-wiring.

In addition to changes in cyclin E-Cdk complex composition, ablation of Cdk2 led to the loss of cyclin E1 binding to p107 (Fig 4A and 4B). Hence, Cdk2 is required for cyclin E1-p107 interaction, and other Cdks cannot replace Cdk2 in this process.

To the best of our knowledge, our quantitative proteomic analysis of Cdk2^-/-^ organs described above represents the first unbiased, proteome-wide study of how cells re-wire protein-protein interactions upon a particular genetic insult.

### Cyclin E1 interactomes are enriched for Cdk2 substrates

Since cyclins are known to physically interact with cyclin-Cdk phosphorylation substrates [19], we hypothesized that mining of the cyclin E1 interactome might allow us to identify new, possibly tissue-specific substrates of cyclin E-Cdk2 kinase. To test this prediction, we first intersected the cyclin E1 interactome with the list of Cdk2 substrates at PhosphoSitePlus (Cell Signaling Technology). Among cyclin E1 interactors we observed several well-established Cdk2 phosphorylation substrates, including p107, p130, p27^Kip1^, Mcm proteins (Mcm3 and Mcm4), Cdc6 and Brca2 (Fig 2A and S1 Table). In total, fifteen out of 117 interactors (12.8%) represented known Cdk2 substrates, compared with 1.95% in the whole proteome (p = 8.9 x 10^−9^) (Table 1).

**Table 1:** Known Cdk2 substrates in the cyclin E1-interactome. The fraction of known Cdk2 substrates among cyclin E1-interactors identified in this study was determined by overlaying our cyclin E1 interactome with the list of Cdk2 substrates from the PhosphoSitePlus database (Cell Signaling Technology), and compared against the fraction of Cdk2 substrates in the entire proteome present in the database. P-value was calculated using one-tailed Fisher’s exact test.

	Cdk2 Substrate		Total	Cdk2 Substrate
	Yes	No		% Total
E1 interactome	15	102	117	12.82
Whole proteome	390	19,609	19,999	1.95

p = 8.9 x 10^−9^

We next screened the amino acid sequence of all 117 cyclin E1 interactors with a Scansite 3.0 program [20]. This software allows one to identify proteins containing a Cdk phosphorylation motif, which hence might represent *bona fide* Cdk2 phosphorylation substrates. We found that 39/117 (33.3%) proteins contained high-confidence and 70/117 (59.8%) medium-confidence Cdk phosphorylation motifs, again a very strong enrichment as compared to the whole proteome (p = 2.4 x 10^−15^ for high-confidence and p < 2.2 x 10^−16^ for medium-confidence substrates) (Table 2 and S2 Table). We concluded that the cyclin E1 interactome likely contains several novel Cdk2 substrates. Gene Ontology analysis of predicted Cdk2 substrates revealed strong enrichment for cell cycle (p = 1.5 x 10^−17^) and transcriptional functions (p = 5.0 x 10^−4^, S2 Table), indicating that cyclin E-Cdk2 kinase influences these functions *in vivo*.

**Table 2:** Predicted Cdk2 substrates in the cyclin E1-interactome. The fraction of predicted Cdk substrates among cyclin E1-interactors identified in this study, versus in the entire proteome present in mouse SwissProt database. Cdk substrates were predicted using Scansite 3.0 using either high-stringency scoring (high-confidence; median score = 0.252) or medium-stringency scoring (med-confidence; median score = 0.341). Median score in the search of the entire proteome was 0.536, indicating even less stringent criterion than the above medium-stringency search. The fractions were compared using one-tailed Fisher’s exact test.

	Predicted Cdk2 Substrate		Total	Cdk2 Substrate
	Yes	No		% Total
E1 interactome High-confidence	39	78	117	33.33
E1 interactome Med-confidence	70	47		59.83
Whole proteome	1,296	15,112	16,408	7.9

High-confidence: p = 2.4 x 10^−15^

Med-confidence: p < 2.2 x 10^−16^

Analyses of individual organ-specific interactomes revealed that in all organs except for testes, approximately one third of proteins represented predicted high confidence Cdk2 phosphorylation substrates, whereas approximately half corresponded to medium confidence substrates. In contrast, in testes this proportion was significantly higher (43.3% and 66.7%, respectively, Table 3), raising a possibility that cyclin E-Cdk2 kinase plays particularly important functions in this compartment. We further explored this possibility in our mechanistic studies of testicular interactome described below.

**Table 3:** Predicted Cdk2 substrates in the organ-specific cyclin E1-interactome. The fraction of predicted Cdk substrates among interactors of cyclin E1 in the indicated organs. The lists of interactors in each organ were analyzed with Scansite 3.0, using high-stringency or medium-stringency scoring.

	Emb Brain	Spleen	Testis	Thymus	Adult Brain
Total interactome	75	49	30	19	28
High-confidence	25 (33.3%)	16 (32.7%)	13 (43.3%)	7 (36.8%)	9 (32.1%)
Med-confidence	44 (58.7%)	27 (55.1%)	20 (66.7%)	11 (57.9%)	14 (50.0%)

### Cyclin E1-Cdk2 kinase phosphorylates components of the DREAM complex

**Fig 5:**
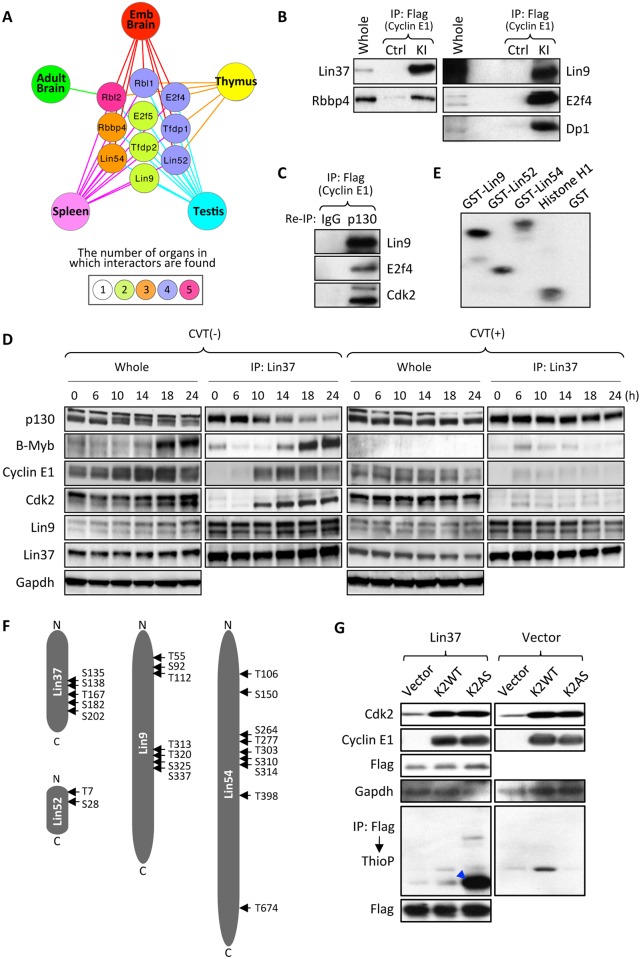
Interaction of cyclin E1 with the DREAM complex. (A) A diagram showing in which organ a particular component of the DREAM complex was identified as a cyclin E1-interacting protein by our mass spectrometric analyses. The number of organs in which a given protein was found to associate with cyclin E1 is depicted by the color. (B) Association of cyclin E1 with components of the DREAM complex in the spleens of tagged knock-in mice (KI) was verified by immunoprecipitating (IP) cyclin E1 with anti-Flag antibody followed by immunoblotting with the indicated antibodies. (C) IP followed by re-IP-immunoblotting to demonstrate that cyclin E1, Cdk2 and the DREAM complex components are present within the same multi-protein complex. Cyclin E1 was immunoprecipitated from spleens of KI mice using anti-Flag antibody, protein complexes were eluted with Flag peptides, re-immunoprecipitated with IgG (control) or with anti-p130 antibody, and then immunoblotted with the indicated antibodies. (D) T98G cells were serum starved for 72 hrs (0% FBS). Subsequently, cells were stimulated to re-enter the cell cycle by addition of 20% FBS supplemented with either 0.2% DMSO (control, left two panels) or 20 μM CVT-313 (right two panels), and harvested at the indicated time-points. Cell extracts (whole) as well as anti-Lin37 immunoprecipitates were resolved on 4–15% gradient SDS-PAGE gels and probed with indicated antibodies. Gapdh serves as a loading control. (E) Cyclin E1-Cdk2 kinase can phosphorylate purified recombinant Lin proteins *in* vitro. Lin9, Lin52 and Lin54 were expressed as GST-fusion proteins in *E*. *Coli*, purified and subjected to *in vitro* kinase reactions with the recombinant cyclin E1-Cdk2 in the presence of [γ^32^P]ATP. Recombinant histone H1 was used as a positive control and GST as a negative control. (F) A diagram illustrating amino acid residues in human Lin proteins that were phosphorylated by cyclin E-Cdk2. (G) Wild-type Cdk2 (K2WT) or analog-sensitive Cdk2 (K2AS) were transfected into 293T cells together with cyclin E1 and Flag-tagged Lin37 or vector control. After supplementing cells with 6-Fu-ATPγS, labeling of Lin37 was evaluated by immunoprecipitating Lin37 with an anti-Flag antibody followed by immunoblotting with anti-thiophosphate ester antibody. A blue arrowhead indicates 6-Fu-ATPγS-labeled Lin37.

Our analyses of cyclin E1 interactomes from different organs revealed that cyclin E1 associates with components of the DREAM complex in several proliferating tissues (Figs 2 and 5A and S1 Table). The DREAM complex represents a group of proteins that have been conserved between *C*. *Elegans*, *Drosophila* and humans [21,22]. In mammalian cells, the DREAM complex consists of a ‘pocket’ protein (p107 or p130), transcription factors E2f4 or E2f5 and Dp1 or Dp2, and five proteins homologous to products of the *C*. *Elegans* synMuvB group of genes: Lin9, Lin37, Lin52, Rbbp4 (Lin53) and Lin54 [22]. The mammalian DREAM complex physically binds to E2f responsive promoters in quiescent cells and represses their transcription. During G_0_ → S phase progression, the Lin subunits dissociate from the p130-E2f-Dp module through an unknown mechanism, and they form a new DNA-binding complex with Myb transcription factor [22].

We first verified the interaction of cyclin E1 with Lin proteins and other components of the DREAM complex in mouse spleens by IP-western blotting (Fig 5B). Although we did not detect Lin37 as cyclin E1 interactor in our mass spectrometry analysis, IP-western blot analysis confirmed that also this DREAM complex subunit associated with cyclin E1 (Fig 5B, S4A and S4B Fig).

Next, we performed immunoprecipitation followed by re-IP and western blotting to verify that cyclin E1, p130 and Lin proteins are present within the same complex (Fig 5C). We also determined that cyclin E1 associates with Lin proteins and with other components of the DREAM complex in mouse embryonic fibroblasts (MEFs) (S4A Fig) and in human glioblastoma T98G cells (S4B Fig). Importantly, when we used for immunoprecipitation an anti-Lin52 antibody which recognizes only ‘free’ Lin proteins, but fails to bring down Lin52 associated with p130, E2f and Dp because its epitope overlaps with the p130-binding site in Lin52 [23,24], we found that this pool of ‘free’ Lin proteins does not associate with cyclin E1 (S4B Fig). These findings indicate that cyclin E1 binds to the DREAM complex through the p130-E2f-Dp module. Consistent with this notion, interaction of cyclin E1 with the DREAM complex was abrogated in knockout MEFs lacking p107, p130 and pRB (S4C Fig).

To determine when during cell cycle progression cyclin E1-DREAM interaction takes place, we synchronized T98G cells by serum starvation and then forced them to re-enter the cell cycle by addition of serum. We found that cyclin E1 associates with Lin37 starting at approximately 10 hrs post-stimulation; the onset of cyclin E1-Lin37 binding coincided with decreased interaction of Lin37 with p130 and preceded association of Lin37 with B-Myb (Fig 5D). This timing suggested that cyclin E1 might play a role in disrupting the DREAM complex.

We determined that cyclin E1 kinase partner Cdk2 also associated with the DREAM proteins (Fig 5C), with kinetics essentially identical to cyclin E1-DREAM interaction (Fig 5D) suggesting that cyclin E-Cdk2 might phosphorylate components of this complex and trigger its disassembly. To test this prediction, we synchronized T98G cells as above and released them in the presence of a CDK2 inhibitor, CVT-313. Indeed, inhibition of CDK2 kinase prevented dissociation of Lin37 from p130 (Fig 5D). Strikingly, CVT313-treatment also prevented binding of cyclin E1 and Cdk2 to Lin37, indicating that an active Cdk2 kinase is needed for cyclin E1/Cdk2-DREAM interaction (Fig 5D).

Next, we ectopically expressed cyclin E1 and Cdk2 together with p130 in T98G cells. We found that expression of cyclin E-Cdk2 led to disruption of the p130-Lin complex (S4D Fig). Significantly, cyclin E1-Cdk2 was also able to decrease binding of Lin subunits to phosphorylation-deficient p130 mutant in which all Cdk phosphorylation sites have been mutated to alanines [25], suggesting that phosphorylation of Lin components by cyclin E1-Cdk2 might contribute to disruption of the p130-Lin complex (S4D Fig). Consistent with this hypothesis, analyses of the cyclin E1 interactome with Scansite 3.0 (S2 Table) indicated that, in addition to a well established Cdk2 substrate p130, four additional components of the DREAM complex, Lin9, Lin37, Lin52 and Lin54 represented predicted Cdk phosphorylation substrates.

To test whether these proteins are indeed phosphorylated by cyclin E-Cdk2, we incubated purified recombinant Lin9, Lin37, Lin52 and Lin54 with cyclin E1-Cdk2 kinase in the presence of radioactive ATP. We found that cyclin E1-Cdk2 readily phosphorylated these proteins *in vitro* (Fig 5E). Subsequently, we used mass spectrometry to map residues on Lin proteins that are phosphorylated by cyclin E1-Cdk2 (Fig 5F and S5 Fig). We extended these observations *in vivo* using cells engineered by us to ectopically express ‘analog-sensitive’ Cdk2 together with cyclin E1 and Flag-tagged Lin37. Unlike wild-type kinases, analog-sensitive kinases can use ‘bulky’ N6-substituted ATP-analogs such as N6-furfuryl-ATP (6-Fu-ATP) to phosphorylate their substrates. Therefore, by supplementing cells expressing analog-sensitive kinases with thio-containing 6-Fu-ATP (6-Fu-ATPγS), one can label their substrates with thiophosphate moieties [26,27]. IP with anti-Flag antibody followed by immunoblotting with anti-thio-phosphate antibody revealed that cyclin E-Cdk2 kinase indeed phosphorylates Lin37 *in vivo* (Fig 5G).

Collectively, these findings suggest that cyclin E-Cdk2 kinase may play an important role in disrupting the DREAM complex during G_0_ → S phase progression, likely through phosphorylation of multiple DREAM subunits.

### Cyclin E1-Cdk2 kinase phosphorylates key spermatogenic transcription factors

We next focused our attention on the cyclin E1 testicular interactome. As mentioned above, genetic ablation of the E-type cyclins led to severe defects in spermatogenesis [3,5]. Strikingly, mice lacking cyclin E kinase partner, Cdk2, displayed a very similar phenotype with spermatocytes arrested at the pachytene stage [6,7]. The molecular basis of this phenotype remains unknown. We hypothesized that cyclin E plays a Cdk2-dependent function in testicular development, through phosphorylation of key proteins that drive the spermatogenic process.

**Fig 6:**
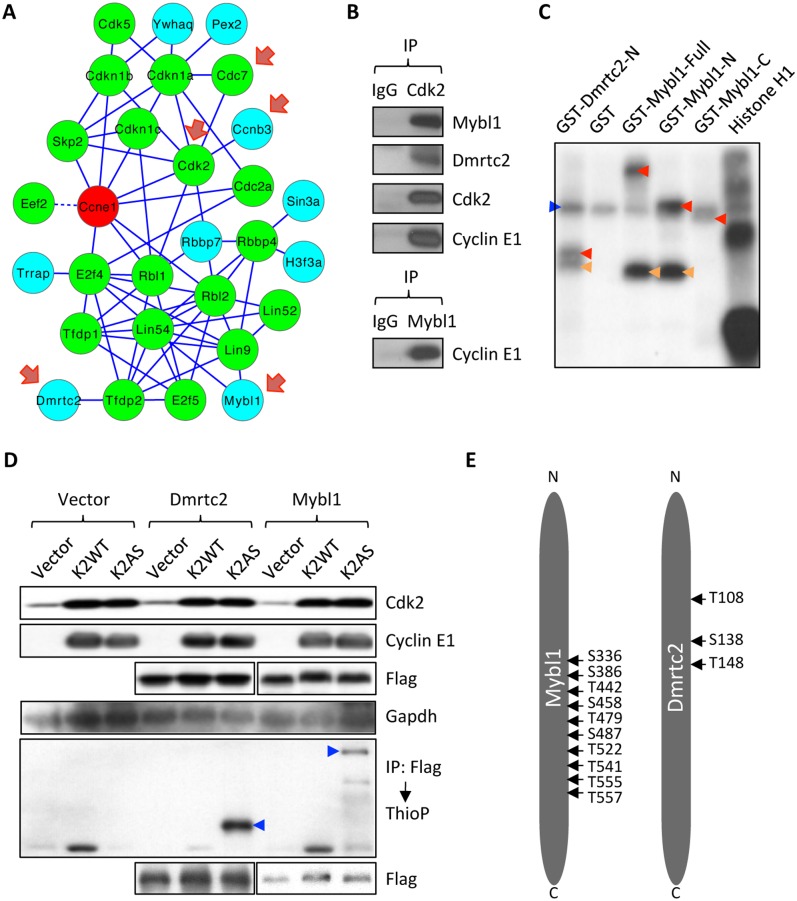
Identification of Mybl1 and Dmrtc2 as cyclin E-Cdk2 phosphorylation substrates in the testes. (A) A diagram illustrating cyclin E1-interactome in testes, consisting of highest-confidence ‘core’ interactors (green nodes), and lower-confidence Category 3 interactors (blue nodes) that were included to the interactome based on their reported interaction with core interactors in the STRING database (see S1 Appendix). Solid lines depict STRING-verified interactions. Dashed lines depict an interaction derived from our mass spectrometry analyses between cyclin E1 and a protein that has no known interactions with other ‘core’ interactors. Red arrows indicate proteins that were previously implicated to play important roles in spermatogenesis. (B) Interaction between endogenous Cdk2/cyclin E and Mybl1 and Dmrtc2 in mouse testes, detected by IP–western blotting. Cdk2 or Mybl1 were immunoprecipitated from lysates of testes, and immunoblots were probed with the indicated antibodies. (C) N-terminal fragment (aa 1–201) of Dmrtc2, as well as full length, N-terminal (aa 1–376), and C-terminal (aa 376–752) fragments of Mybl1 were expressed as GST-fusion proteins in *E*. *Coli*, purified and subjected to *in vitro* kinase reactions with the recombinant cyclin E1-Cdk2 in the presence of [γ ^32^P]ATP. Recombinant histone H1 was used as a positive control and GST as a negative control. Red arrowheads point to phosphorylated GST-fusion proteins, orange arrowheads indicate phosphorylated truncated proteins, and blue arrow indicates auto-phosphorylated recombinant cyclin E1-Cdk2. (D) Wild-type Cdk2 (K2WT) or analog-sensitive Cdk2 (K2AS) were transfected into 293T cells together with cyclin E1 and Flag-tagged substrates (Mybl1 or Dmrtc2). After supplementing cells with 6-Fu-ATPγS, labeling of substrates was evaluated by immunoprecipitating Mybl1 or Dmrtc2 with anti-Flag antibody followed by immunoblotting with an anti-thiophosphate ester antibody. Blue arrowheads indicate ATPγS-labeled Mybl1 and Dmrtc2. The experiment was performed on the same gel as the one shown in Fig 5G, hence the vector control (Vector) is identical. (E) A diagram illustrating amino acid residues in Mybl1 and Dmrtc2, which were phosphorylated by cyclin E-Cdk2.

Our analyses of the testicular cyclin E1 interactome revealed the presence of several proteins that have been shown to play important roles in spermatogenesis, notably Mybl1, Dmrtc2, Cdc7 and cyclin B3, in addition to Cdk2 (Fig 6A and S1 Table). In particular, Mybl1 is thought to represent a ‘master regulator’ of male meiosis [28]. Intriguingly, mice lacking Mybl1 show defective spermatogenesis, which resembles the phenotype of Cdk2- or cyclin E-deficient mice [28,29]. Dmrtc2 is also essential for male spermatogenesis, and Dmrtc2-deficient mice show a similar testicular phenotype with spermatogenic arrest at the pachytene stage [30,31]. Importantly, our Scansite 3.0 analyses of the testicular interactome identified both Mybl1 and Dmrtc2 as predicted Cdk phosphorylation substrates (S2 Table). These findings suggested that Mybl1 and Dmrtc2 might represent essential downstream targets of cyclin E-Cdk2 kinase in testes. Consistent with this possibility, we confirmed the physical interaction between the endogenous Mybl1 and Dmrtc2 proteins and cyclin E and Cdk2 in the mouse testes, by IP–western blotting (Fig 6B).

To test whether Mybl1 and Dmrtc2 can be phosphorylated by cyclin E-Cdk2, we performed *in vitro* kinase assays using recombinant proteins. Indeed, we detected phosphorylation of both proteins by cyclin E-Cdk2 (Fig 6C). We next examined *in vivo* phosphorylation of Mybl1 and Dmrtc2 by cyclin E-Cdk2 using cells engineered by us to express Flag-tagged Mybl1 or Dmrtc2 together with cyclin E and analog-sensitive Cdk2. Immunoprecipitation with anti-Flag antibody followed by western blot analysis with anti-thio-phosphate antibody revealed that cyclin E-Cdk2 kinase indeed phosphorylates Mybl1 and Dmrtc2 *in vivo* (Fig 6D). We next identified residues of Mybl1 and Dmrtc2 that are phosphorylated by cyclin E-Cdk2 using mass spectrometry. A total of ten cyclin E-Cdk2 phosphorylation sites in Mybl1 and three sites in Dmrtc2 were detected (Fig 6E and S6 Fig).

**Fig 7:**
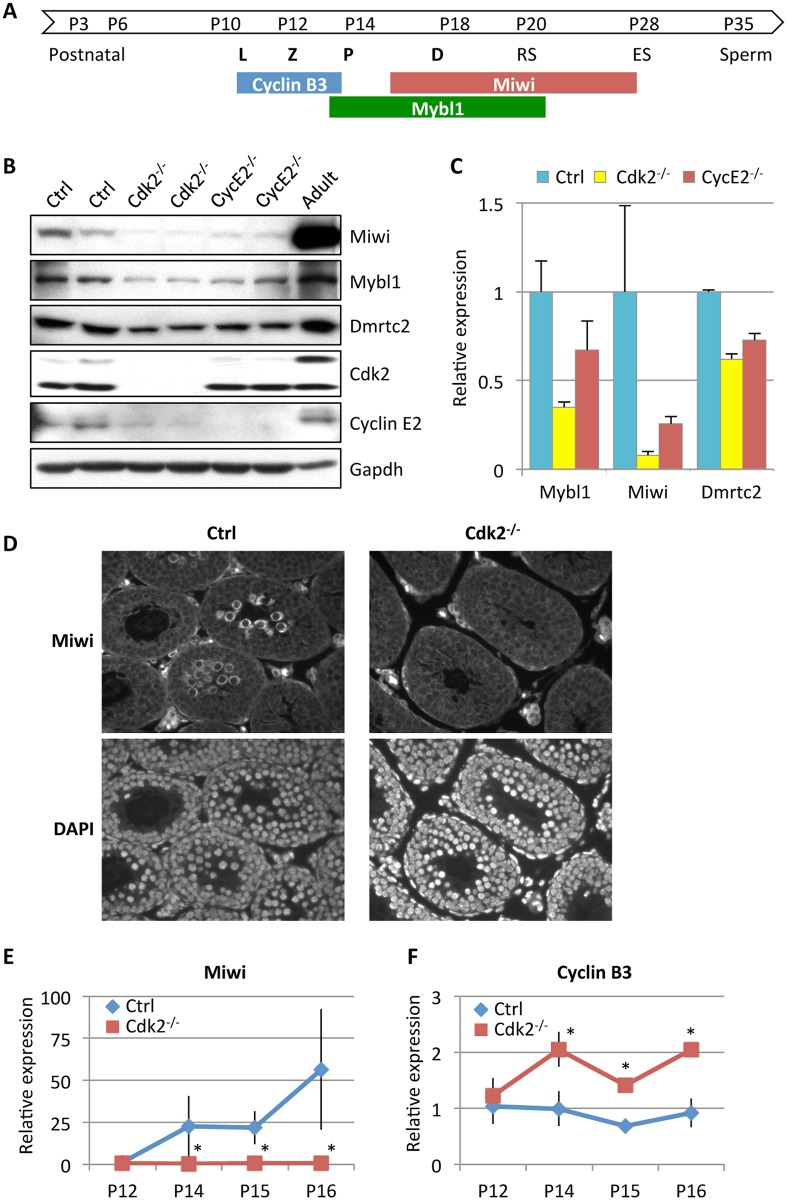
Reduced levels of Mybl1 and Dmrtc2 and altered levels of Mybl1 transcriptional targets in Cdk2- and cyclin E-deficient testes. (A) Schematic representation of expression patterns for Mybl1, Miwi, and cyclin B3 during spermatogenesis. White arrow shows progression of mouse spermatogenesis after birth (P, postnatal days). Meiotic phases (L, leptotene; Z, zygotene; P, pachytene; D, diplotene) are indicated in bold. RS, round spermatids; ES, elongating spermatids. (B) Levels of Miwi, Mybl1 and Dmrtc2 in the testes of P15 wild-type (Ctrl), *Cdk2*^*-/-*^ and *cyclin E2*^*-/-*^ mice were detected by immunoblotting. Protein lysates from testes of 1-month-old wild-type mice were used as a positive control (Adult). (C) Quantification of Mybl1, Miwi and Dmrtc2 levels shown in (B). Data were normalized to Ctrl and represent mean ± SD. (D) Sections of seminiferous tubules obtained from testes of P15 wild-type (Ctrl) and *Cdk2*^*-/-*^ mice were stained with anti-Miwi antibody followed by Alexa 568. Lower panels: DAPI staining to visualize cell nuclei. (E and F) RT-qPCR analyses to gauge levels of Miwi (E) and cyclin B3 (F) transcripts at the indicated postnatal days (P12–P16) in the testes of wild-type (Ctrl) or *Cdk2*^*-/-*^ mice. Error bars represent SD (n = 4 for Ctrl; n = 5 for *Cdk2*^*-/-*^ at each time-point). *p<0.05 using unpaired *t* test.

The essential role of Mybl1 in spermatogenesis is thought to be mediated by the ability of this transcription factor to regulate expression of crucial meiotic genes such as Miwi and Morc2b. Indeed, disruption of these Mybl1 downstream targets can also cause abnormal spermatogenesis [28]. During the course of normal spermatogenesis, Mybl1 expression increases at the pachytene stage, leading to increased transcription of Mybl1 downstream targets such as Miwi (Fig 7A). Strikingly, in *Cdk2*^*-/-*^ mice the expression of Mybl1 remained low (Fig 7B and 7C). Consequently, Cdk2-null testes failed to express normal levels of Miwi, a rate-limiting transcriptional target of Mybl1, as revealed by reverse transcription–quantitative PCR (RT-qPCR), western blotting and immunohistochemistry (Fig 7B–7E). These results suggest that phosphorylation of Mybl1 by cyclin E-Cdk2 is required to stabilize and activate Mybl1 at the pachytene stage.

In addition, cyclin B3 also represents a direct transcriptional target of Mybl1. Unlike the majority of Mybl1 target genes, where Mybl1 serves as an activator of transcription, expression of cyclin B3 is repressed by Mybl1 [28]. Cyclin B3 is highly expressed in pre-pachytene spermatocytes and downregulated when cells enter the pachytene stage and start expressing Mybl1 [32] (Fig 7A). Aberrant expression of cyclin B3 beyond the pachytene stage causes spermatogenic defects [33]. Consistent with reduced Mybl1 levels observed by us in Cdk2-null testes, we found that expression of cyclin B3 was elevated in the absence of Cdk2 (Fig 7F). These results suggest that cyclin E-Cdk2 negatively regulates expression of cyclin B3 through Mybl1, and that deregulated cyclin B3 expression also likely contributes to the testicular phenotype seen in Cdk2-null testes.

DNA-binding protein Dmrtc2 represented another testicular-specific substrate of cyclin E1-Cdk2 kinase identified in our analyses (Fig 6B–6D). Dmrtc2 is an essential regulator of spermatogenesis, and mice lacking this protein present impairment in spermatogenesis characterized by developmental arrest at the pachytene stage [30,31]. However, the molecular function of Dmrtc2 in spermatogenesis is not yet well understood. Western blotting of Cdk2-null testes revealed that ablation of Cdk2 led to a reduction of Dmrtc2 protein levels (Fig 7B and 7C). We propose that cyclin E-Cdk2 kinase may also affect spermatogenesis via Dmrtc2, by regulating the levels and function of Dmrtc2, through direct phosphorylation.

Of the two cyclin E proteins, cyclin E2 is more abundant than cyclin E1 in mouse testes [5,34]. Correspondingly, cyclin E2-null mice manifest more evident spermatogenic defects and decreased fertility, although the abnormalities are not as pronounced as those seen in Cdk2-null mice, due to compensation from cyclin E1 in E2-null cells [3,5]. Despite these differences in phenotypic severity, *cyclin E2*^*-/-*^ testes showed very similar aberrant expression of Mybl1, Miwi and Dmrtc2 to that seen Cdk2-null mice (Fig 7B and 7C). Low expression of Miwi that persists in cyclin E2-null spermatocytes indicates low residual activity of Mybl1, which is likely caused by phosphorylation of Mybl1 by cyclin E1-Cdk2, and may explain why *cyclin E2*^*-/-*^ mice have milder testicular phenotype than Cdk2-null animals.

Taken together, these results strongly suggest that cyclin E-Cdk2 kinase plays a critical role in the male germline by phosphorylating and controlling the activity of key regulators of spermatogenesis Mybl1 and Dmrtc2.

## Discussion

In this study we used knock-in mice expressing tandemly tagged cyclin E1 in place of the wild-type protein to delineate the proteomic landscape of cyclin E1 interactions *in vivo*. When combined with large-scale mass spectrometry, tagged knock-in mouse system allows one to determine the set of cyclin E1 interacting proteins (E1-interactome) in essentially any organ or cell type. We also demonstrate that this system can be used to identify novel, tissue-specific substrates of cyclin E-Cdk2 kinase. Moreover, by using quantitative proteomic approaches, one can visualize how the cyclin E1 interactome changes under different physiological or pathological conditions. As a proof of principle, we bred our tagged knock-in mice into the Cdk2-null background, and demonstrated re-wiring of the cyclin E1 interactome upon ablation of the major catalytic partner of cyclin E1. In the future, the same approach can be used to determine how the set of cyclin E1-interating proteins or cyclin E-Cdk2 phosphorylation substrates changes in any genetic background and upon any genetic insult. Furthermore, our system allows to visualize and to quantify how the cyclin E1-interactome changes at different stages of normal development or in different pathological states (for example, by comparing young versus aging stem cell compartments). It can also be used to study cyclin E function at different stages of the neoplastic process. Given the well-documented role of cyclin E1 in oncogenesis [35], it will be of interest to compare cyclin E1-interactomes and cyclin E1-Cdk2 phosphorylation targets in pre-malignant lesions, during tumor initiation, progression, and in the metastatic spread.

Our study illustrated that upon ablation of Cdk2, cyclin E1 binds several cell cycle kinases. Such re-wiring of interactomes likely takes place in several mouse knockout strains and compensates for the loss of a given protein. These observations underscore the fact that the experiments using knockout mice should not be over-interpreted, and absence of phenotypes does not rule out a physiological role for a given protein.

Our proteomic analyses revealed that regulation of cell cycle and transcription represents the major biological functions regulated by cyclin E1 (and E1-Cdk2 kinase) *in vivo*. The role of cyclin E in cell cycle progression has been extremely well documented. Several studies also implicated cyclin E1 in transcription [36–42]. Our analyses indicate that cyclin E plays a rate-limiting role in regulating transcription *in vivo* in the male germline, where it controls expression of key meiotic genes via Mybl1 (and likely Dmrtc2).

We found that cyclin E-Cdk2 kinase phosphorylates a master regulator of spermatogenesis, Mybl1, and that the levels of Mybl1 are decreased in Cdk2- and cyclin E2-null testes. Moreover, the expression of Mybl1 transcriptional targets (such as Miwi and cyclin B3) was deregulated, consistent with the loss of Mybl1 activity. Intriguingly, mice lacking cyclin E, Cdk2, Mybl1, or Miwi share similar spermatogenic defects [3,5–7,28,29]. We propose that cyclin E-Cdk2 kinase serves to maintain transcriptional activity of Mybl1 in the male germline. It remains to be seen how exactly cyclin E-Cdk2 regulates Mybl1 function. The absence of faithful *in vitro* systems to study meiotic cells precluded us from performing mechanistic analyses to address this point.

Dmrtc2 represents another critical protein implicated in mouse spermatogenesis, and spermatocytes lacking Dmrtc2 exhibit pachytene arrest, similar to testicular phenotype seen in Cdk2- or cyclin E-deficient animals [30,31]. We found that, like Mybl1, Dmrtc2 also represents a direct cyclin E-Cdk2 phosphorylation target, and that the levels of Dmrtc2 are reduced in the testes of mice lacking Cdk2 or cyclin E. Collectively, these results suggest that cyclin E-Cdk2 represents a crucial upstream regulator of the transcriptional cascade in the male germline, by acting through Mybl1 and Dmrtc2.

Analyses of cyclin E interactomes in embryonic and adult brains suggest novel, previously unanticipated functions for cyclin E in neurogenesis and in regulation of microtubule-based processes and microtubule cytoskeleton. These findings will now allow one to design hypothesis-driven studies to elucidate cyclin E functions in these processes, based on the interactors identified in our study. Cyclin E is highly expressed in adult mouse brains, where it was shown to regulate synaptic plasticity by inhibiting phosphorylation of synaptic Cdk5 substrates [8]. However, very little is known about the function of cyclin E in regulating neuronal cytoskeleton and in neurogenesis. Previous work in Drosophila revealed a role for cyclin E in fate determination in the central nervous system. Loss of cyclin E function in the developing neuroblast lineage was shown to result in generation of only glial cells, while ectopic expression of cyclin E led to generation of neuronal sublineage, in addition to the glial cells [43]. This role of cyclin E was attributed to regulation of localization and function of a homeobox protein Prospero [44]. Given results of our proteomic analyses, it seems likely that cyclin E affects neuronal differentiation also in mammalian cells via currently unknown mechanism(s).

The study described here focused on a key component of mammalian core cell cycle machinery, which has been conserved from yeast to humans. Our results indicate that while preserving their ‘core’ cell cycle functions, in the process of evolution these proteins acquired novel, tissue-specific roles. In the future, the same approach can be applied to study the function of any protein in any model organism. By combining and overlaying interactomes of various interacting proteins (for example kinases and all their regulatory and accessory partners) one will be able to visualize complex biological networks that control, in a cell type-specific fashion, different cellular functions. Unbiased, biocomputational analyses of these networks will help to understand the biological interplay between different proteins, and to elucidate how perturbations of components of these networks contribute to various pathological states.

## Materials and Methods

### Generation of tagged cyclin E1 knock-in mice and mouse crosses

Detailed procedures to generate knock-in mice carrying a Flag-HA tag at the N-terminus of the *cyclin E1* gene (cyclin *E1*^*Ntag/Ntag*^) have been described previously [8]. A targeting vector to knock-in a Flag-HA tag at the C-terminus of the *cyclin E1* gene was constructed by replacing a stop codon in the last exon with DNA sequences encoding a Flag-HA tag followed by a termination codon, and by inserting a *loxP*-flanked hygromycin (Hyg) resistance cassette into ScaI site in the intron 11 (Fig 1B). The construct spanned 10 kb KpnI–HpaI fragment of the *cyclin E1* gene. The targeting vector was electroporated into embryonic stem (ES) cells and homozygous *cyclin E1*^*Ctag/Ctag*^ animals were obtained using standard procedures [45]. *Cyclin E1*^*Ntag/Ntag*^ mice were crossed with *Cdk2*^*+/-*^ animals (kindly provided by Dr. Philipp Kaldis). All experiments conformed to the relevant regulatory standards, and were approved by the Institutional Animal Care and Users Committee.

### Purification of cyclin E1-containing complexes from mouse organs and mass spectrometry

Spleens, testes, thymuses, and brains were dissected from 1-month-old *cyclin E1*^*Ntag/Ntag*^ or cyclin *E1*^*Ctag/Ctag*^ mice. Embryonic brains (heads) were collected from E14.5–15.5 embryos. We used pooled 20 to 30 adult organs or 20 embryonic heads for a single purification. To maximize the capture of interactors, we used approximately 1:1 mixture of organs derived from amino- and carboxy-tagged mice. After homogenizing tissues, cyclin E1 and its associated proteins were immunoprecipitated using anti-Flag M2 agarose (Sigma), eluted twice with Flag peptide (Sigma), then immunoprecipitated again with anti-HA antibody (12CA5 ascites fluid, Covance) coupled to protein A sepharose beads (Amersham). Complexes were then eluted with 0.1 M glycine (pH 2.5). For mass spectrometry, purified protein complexes containing at least 200–300 ng of cyclin E1 were used for a single run. For each organ, we performed 6–10 purifications (each yielding 200–300 ng of cyclin E1), followed by 6–10 independent mass spectrometry runs. In parallel, we performed 6–11 ‘mock’ purifications from the same number of organs from wild-type mice, followed by 6–11 mass spectrometry runs. Detailed procedures for LC-MS/MS and iTRAQ have been described previously [8].

### Proteomic data analyses and phosphorylation site mapping

Please see S1 Appendix. LC-MS analyses of phosphorylated peptides were performed as previously [46].

### Protein extraction and immunoblot analyses

Organs or cells were homogenized in lysis buffer (50 mM Tris-HCl pH 7.4, 150 mM NaCl, 0.5% NP-40, 10 mM NaF, and protease inhibitor cocktail). Proteins were separated on SDS-PAGE gels and transferred to Immobilon-P membranes (Millipore). Membranes were blocked with blocking buffer (TBST, 5% non-fat skim milk) before immunoblotting. Immunoblots were visualized by ECL (Pierce) or Odyssey imaging system (LI-COR). Quantification was performed with ImageJ. For immunoprecipitation, organs or cells were homogenized in lysis buffer containing 100 mM Tris-HCl pH 8.0, 100 mM KCl, 0.1% NP-40, 0.1% Tween 20, 10 mM NaF, and protease inhibitor cocktail.

### Immunostaining

Testes were collected from wild-type and *Cdk2*^*-/-*^ mice at P15, fixed in 4% paraformaldehyde, and embedded in paraffin. Section (5 μm-thick) were deparaffinized through xylene and graded ethanol dilutions, followed by antigen retrieval by microwaving in PBS buffer (5 mM Tris pH 8.0, 1 mM EDTA). After blocking with 5% normal goat serum (NGS; Sigma) and 0.2% Triton X-100 in PBS for 1 hr at RT, sections were incubated with primary antibodies for 2 hrs in PBS containing 5% NGS and 0.2% Triton-X100 at RT, washed with PBS, then incubated with secondary antibodies (Alexa 568 and Alexa 488; Invitrogen) for 1 hr at RT in PBS with 5% NGS. After rinsing with PBS, sections were mounted with Vectashield mounting medium containing DAPI (Vector Laboratories) and analyzed on a fluorescent microscope (Nikon E600).

### Antibodies

For immunoblotting, immunoprecipitation and immunostaining, we used antibodies against cyclin E1 (Santa Cruz, BioLegend or Millipore), cyclin D1, cyclin D3, cyclin B1, Cdk1, Cdk2, Cdk4, Cdk5, p107, Pcna, B-Myb, Fig 4, Rbbp9 (Santa Cruz), p130 (Santa Cruz or BD Transduction Labs or Bethyl Laboratories), HA (Covance), actin, Flag, Mybl1, cyclin A2 (Sigma), Gapdh (Invitrogen or Millipore), Lin9, Lin37, Lin52, Lin54, Rbbp4, Rbbp7 (Bethyl Laboratories), E2f4, Dp1, Cdc7 (Lab Vision), thiophosphate ester, Dmrtc2 (Abcam or Sigma), Mapk15 (Abcam), RalA, mTOR/Frap1, cyclin B3, phosphor-Rb, Tubulin and Miwi (Cell Signaling). Purified rabbit IgG was from either Bethyl Laboratories or Santa Cruz and mouse IgG from Santa Cruz. For immunoprecipitation, we also used anti-Flag M2 agarose (Sigma) and HA beads that were prepared by conjugating Protein A Sepharose (Amersham) with anti-HA antibody in 12CA5 ascites fluid (Covance).

### Generation of GST-constructs

To construct GST-Mybl1 and GST-Dmrtc2, cDNA fragments were amplified by PCR using human Mybl1 cDNA and mouse Dmrtc2 cDNA (from Dr. D. Zarkower) as templates and subcloned into pGEX-5X-3 vector (GE Healthcare). GST-Mybl1-N and GST-Mybl1-C contain DNA segments encoding N-terminal (aa 1–376) and C-terminal (aa 376–752) fragments of Mybl1, respectively. GST-Dmrtc2-N contains a DNA segment encoding N-terminal fragment (aa 1–201) of Dmrtc2. To construct GST-Lin9, -Lin37, -Lin52 and -Lin54, corresponding full-length human cDNAs were subcloned into pGEX-6P-3 vector (GE Healthcare). The GST-containing constructs were expressed in *E*. *Coli* BL21, and proteins were purified using glutathione sepharose (GE Healthcare).

### Expression vectors

To construct pCMV-Flag-Mybl1 and pCMV-Flag-Dmrtc2, cDNA fragments were amplified by PCR using human Mybl1 cDNA and mouse Dmrtc2 cDNA (from Dr. D. Zarkower) as templates and subcloned into p3XFLAG-CMV expression vector (Sigma). Wild-type and AS mouse Cdk2 were cloned into pCMV vector, and pCMV-cyclin E was provided by Dr. B. Clurman.

### Cdk2 inhibition in tissue lysate

Protein lysates from spleens collected from wild-type mice were incubated with 20 μM CVT-313 (Santa Cruz Biotechnology) for 30 min at room temperature. Subsequently, lysates were used for immunoprecipitation in the presence of 20 μM CVT-313 inhibitor. Washing buffer also contained 20 μM CVT-313.

### *In vitro* kinase assays

GST-Lin9, GST-Lin37, GST-Lin52, GST-Lin54, GST-Mybl1 and GST-Dmrtc2, were constructed as described above. For kinase assays, GST proteins (1 μg) or histone H1 (1 μg; Roche) were incubated with recombinant cyclin E-CDK2 (1 μg; Millipore) at 30°C for 30 min in 20 mM Tris-HCl pH 8.0, 1 mM EGTA, 10 mM MgCl_2_, 1 mM dithiothreitol, 25 μM cold ATP and 10 μCi [γ^32^P]-ATP. For inhibition of Cdk2 kinase activity, 20 μM CVT-313 was added to both reaction and washing buffer.

### *In vivo* kinase assays

To generate an analog-sensitive (AS) version of Cdk2, we introduced a mutation in mouse Cdk2 cDNA that changes phenylalanine 80 to a glycine as described previously [47]. Human embryonic kidney 293T cells in 6-well plates were co-transfected using lipofectamine 2000 (Invitrogen) with plasmids encoding Flag-tagged Lin37, Mybl1 or Dmrtc2 and AS Cdk2 or wild-type Cdk2 with cyclin E1. After two days, cells were washed with PBS and incubated in the wells for 20 min at room temperature with 200 μl of a kinase reaction buffer [20 mM HEPES pH 7.5, 100 mM KOAc, 5 mM NaOAc, 2 mM MgOAc_2_, 1 mM EGTA, 10 mM MgCl_2_, 0.5 mM DTT, 30 μg/ml digitonin, 5 mM GTP, 0.1 mM ATP, 0.1 mM N6-(phenethyl) ATPγS (Biolog), 1X phosphatase inhibitor cocktail I and II (Sigma), and 1X complete protease inhibitors, EDTA-Free (Roche)], as described previously [26]. After the labeling step, 200 μl of 2x RIPA buffer (100 mM Tris pH 8.0, 300 mM NaCl, 2% NP-40, 0.2% SDS, 20 mM EDTA) with 2.5 mM p-nitrobenzyl mesylate (PNBM; Abcam) was added, and samples were incubated for 30 min at room temperature (RT). Mybl1 and Dmrtc2 were immunoprecipitated using anti-Flag antibody-coupled resin (Sigma) and the phosphorylation was detected by western blotting with an anti-thiophosphate ester antibody (Abcam).

### Quantitative RT-PCR

Testes dissected from wild-type or *Cdk2*^*-/-*^ mice were homogenized in Trizol (Invitrogen), and total RNA was extracted. Reverse transcription and qPCR were performed using SuperScript III SYBR Green One-Step kit (with ROX; Invitrogen). Data were normalized to Gapdh levels, and calculations were made based on the ΔΔCT method.

### Cell culture

All cells were cultured in DMEM with 10% fetal bovine serum (Sigma). Early passage wild type MEFs and TKO (*Rb*^*-/-*^*p107*^*-/-*^*p130*^*-/-*^) MEFs [48] (a gift from H. te Riele) were grown until 80% confluent, harvested and analyzed by immunoprecipitation and immunoblotting.

### Gel filtration

Protein lysates were separated by size exclusion chromatography using a Superdex 200 10/300 GL (GE Healthcare). Approximately 250 μL of samples were loaded onto the Superdex size exclusion column in buffer (50 mM Tris-HCl, pH 8.0).

### Statistical analysis

All statistical analyses were performed by using R or MATLAB.

## Supporting Information

S1 Fig Analyses of the five mouse organs used for proteomic analyses. (A) Sections of adult brains, testes, spleens and thymuses, as well as of embryonic brains, stained for Ki67. (B) Quantification of the fraction of proliferating (Ki67-positive) cells from (A). (C) The levels of Ser807/811-phosphorylated pRB (Phospho-Rb), total pRB, and cyclin A2 in the five mouse organs. Tubulin served as a loading control.
(TIF)

S2 Fig Verification of selected cyclin E1-interactors. (A) Selected cyclin E1-interactors identified in our mass spectrometric analyses were verified by immunoprecipitating cyclin E1 from spleens of tagged cyclin E1 knock-in mice, followed by immunoblotting with the indicated antibodies. (B) Verification of the interaction between endogenous Cdk2 or endogenous wild-type cyclin E1 and selected novel interactors. (C) Verification of the interaction between cyclin E1 and selected novel interactors in human HeLa cells.
(TIF)

S3 Fig Quantitative proteomic (iTRAQ) analysis of cyclin E1-interacting proteins in mouse thymuses in the absence of Cdk2. (A) The amount of cyclin E1-associated Cdk1, Cdk2, Cdk4 and Cdk5 in the thymuses of wild-type (Ctrl), *Cdk2*^*+/+*^/*cyclin E1*^*Ntag/Ntag*^ (KI), and *Cdk2*^*-/-*^*/cyclin E1*^*Ntag/Ntag*^ (*Cdk2*^*-/-*^) mice was gauged by immunoprecipitation with an anti-Flag antibody and immunoblotting with the indicated antibodies. (B) Ablation of Cdk2 does not trigger re-distribution of cyclin-bound Cdk4 and Cdk1. Cyclins D1, A2 or B1 were immunoprecipitated from lysates prepared from wild-type (Ctrl) or Cdk2^-/-^ spleens and immunoblots were probed with the indicated antibodies. Whole, whole cell lysates. Tubulin served as a loading control.
(TIF)

S4 Fig Analyses of the interaction between cyclin E1 and the DREAM complex. (A) Immunoprecipitation (IP) followed by re-IP-immunoblotting to demonstrate that cyclin E1, Cdk2 and the DREAM complex components are present within the same multi-protein complex. Protein lysates from wild-type (Ctrl) or KI mouse embryonic fibroblasts (MEFs) were immunoprecipitated with anti-Flag antibody, eluted with Flag peptide, re-immunoprecipitated with anti-p130 or -Lin9 antibodies, and then immunoblotted with the indicated antibodies. (B) The DREAM complex subunits were immunoprecipitated from human glioblastoma T98G cell extracts with the indicated antibodies and immunoblots were probed with the antibodies against p130, cyclin E1 and Lin9. (C) Extracts prepared from wild-type (WT) and triple-knockout (TKO) Rb^-/-^p107^-/-^p130^-/-^ MEFs lacking pRB, p107 and p130 were immunoprecipitated with an anti-Lin37 antibody. The immunoprecipitates and 10% input (whole cell lysates) were resolved on SDS-PAGE gel and probed with indicated antibodies. (D) T98G cells were transfected with HA-tagged wild-type p130 (WT) or pan-phosphorylation mutant (PM), with or without HA-tagged Cdk2 (HA-Cdk2) and Myc-tagged cyclin E1 (Myc-Cyclin E1), as indicated. Cells were lysed 24 hrs post transfection and immunoprecipitated with an anti-Lin37 antibody or, for control, with IgG. Whole cell lysates (10% of input) and immunoprecipitates were resolved on a SDS-PAGE gel and analyzed by immunoblotting with the indicated antibodies.
(TIF)

S5 Fig Mapping of cyclin E1-Cdk2 phosphorylation sites on Lin proteins by mass spectrometry. Amino acid sequences of human Lin proteins are shown. Peptide fragments that were detected by mass spectrometry are highlighted in yellow, and identified cyclin E-Cdk2 phosphorylation sites are labeled in red.
(TIF)

S6 Fig Mapping of cyclin E1-Cdk2 phosphorylation sites on Mybl1 and Dmrtc2 by mass spectrometry. Amino acid sequences of human Mybl1 and Dmrtc2 are shown. Peptide fragments that were detected by mass spectrometry are highlighted in yellow, and identified cyclin E-Cdk2 phosphorylation sites are labeled in red. Note that the recombinant protein used to examine phosphorylation sites in Dmrtc2 was an N-terminal fragment protein (aa 1–201).
(TIF)

S1 Table Identification of cyclin E1-associated proteins in various mouse organs by mass spectrometry. This table contains six separate worksheets, first five of which show lists of proteins identified in LC-MS/MS analyses from knock-in (KI) or wild-type control (WT) embryonic brains (EB), spleens, testes, thymuses, and adult brains (AB), respectively. Proteins in the ‘core’ (Category 1) group are highlighted in green, and those in Categories 2 and 3 in yellow and blue, respectively. Each row lists protein name, gene name, STRING name (mouse and human), gene ID, the number of peptides found in KI (Peptides KI) and WT (Peptides WT), the number of detections across experiments for KI (KI detected) and for WT (WT detected), p-value tissue, ratio tissue, p-value all, and ratio all (see S1 Appendix for details of statistical tests). The penultimate column identifies proteins (marked “Y”) that were included into the ‘core’ Category 1 group based on their identification as a highest-confidence interactor in another organ (see S1 Appendix). The last column identifies proteins from Categories 2 and 3 (marked “Y”) that were included into the interactome based on known, STRING-verified interaction with at least one of the ‘core’ interactors. The last worksheet lists 117 proteins from the combined cyclin E1 interactome in all organs.
(XLSX)

S2 Table Analyses of cyclin E1-interactors. This table contains three separate worksheets. The first shows the list of proteins present in the cyclin E1 interactome in all organs. For each cyclin E1- interactor, gene name is listed in the first column, and the major Gene Ontology term assigned to generate Fig 3D in the second column. The third column identifies proteins that were predicted as high- or medium-stringency putative Cdk phosphorylation substrates predicted by Scansite 3.0 (marked ‘High’ and ‘Medium’, respectively). Also shown are organs in which a given protein was identified as cyclin E1-interactor. EB, embryonic brain; AB, adult brain. The second (High) and third (Medium) worksheets list Gene Ontology terms enriched among high stringency and medium stringency putative Cdk phosphorylation substrates, respectively.
(XLSX)

S3 Table Biological process/molecular function enrichment heat map of cyclin E1 interactors. This table contains six separate worksheets and lists Gene Ontology terms enriched among cyclin E1-interactors detected in the indicated organs. EB, embryonic brain; AB, adult brain.
(XLSX)

S4 Table iTRAQ quantitative comparison of spleen samples. Each row corresponds to a different protein. Shown are: (A) Accession number; (B) Protein name; (C) Ratio of the relative abundance of a given protein between *cyclin E1*^*Ntag/Ntag*^ purification products (KI) versus mock purification (WT); (D) Ratio of the relative abundance of a given protein between *Cdk2*^-/-^/*cyclin E1*^*Ntag/Ntag*^ purification products (Cdk2KO) versus *cyclin E1*^*Ntag/Ntag*^ purification products (KI); (E) For each protein, Cdk2KO:KI ratio was normalized against the abundance of cyclin E1 in Cdk2KO and KI purification products.
(XLSX)

S5 Table Primers used for RT-qPCR. The table lists the forward and reverse primers used for RT-qPCR (Fig 7E and 7F).
(XLSX)

S1 Appendix Supplemental experimental procedures. (DOCX)
