# Supplementary figures and images for: Proteomic Landscape of Tissue-Specific Cyclin E Functions in Vivo
Source: PLoS Genet. 2016 Nov 9;12(11):e1006429. doi: 10.1371/journal.pgen.1006429 (PMC5102403; doi:10.1371/journal.pgen.1006429)

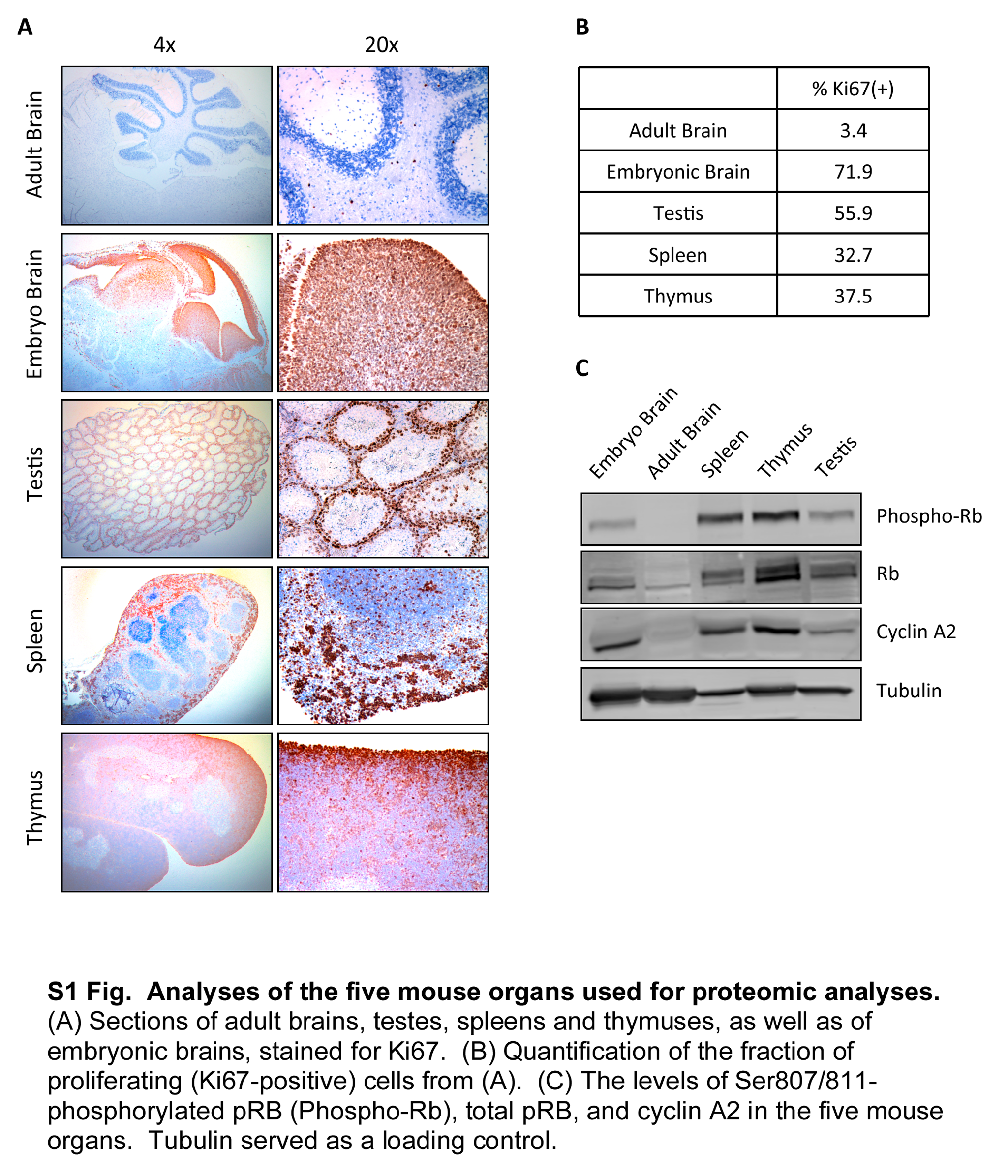

Supplement: S1 Fig — (A) Sections of adult brains, testes, spleens and thymuses, as well as of embryonic brains, stained for Ki67. (B) Quantification of the fraction of proliferating (Ki67-positive) cells from (A). (C) The levels of Ser807/811-phosphorylated pRB (Phospho-Rb), total pRB, and cyclin A2 in the five mouse organs. Tubulin served as a loading control. (TIF) [file pgen.1006429.s001.tif]

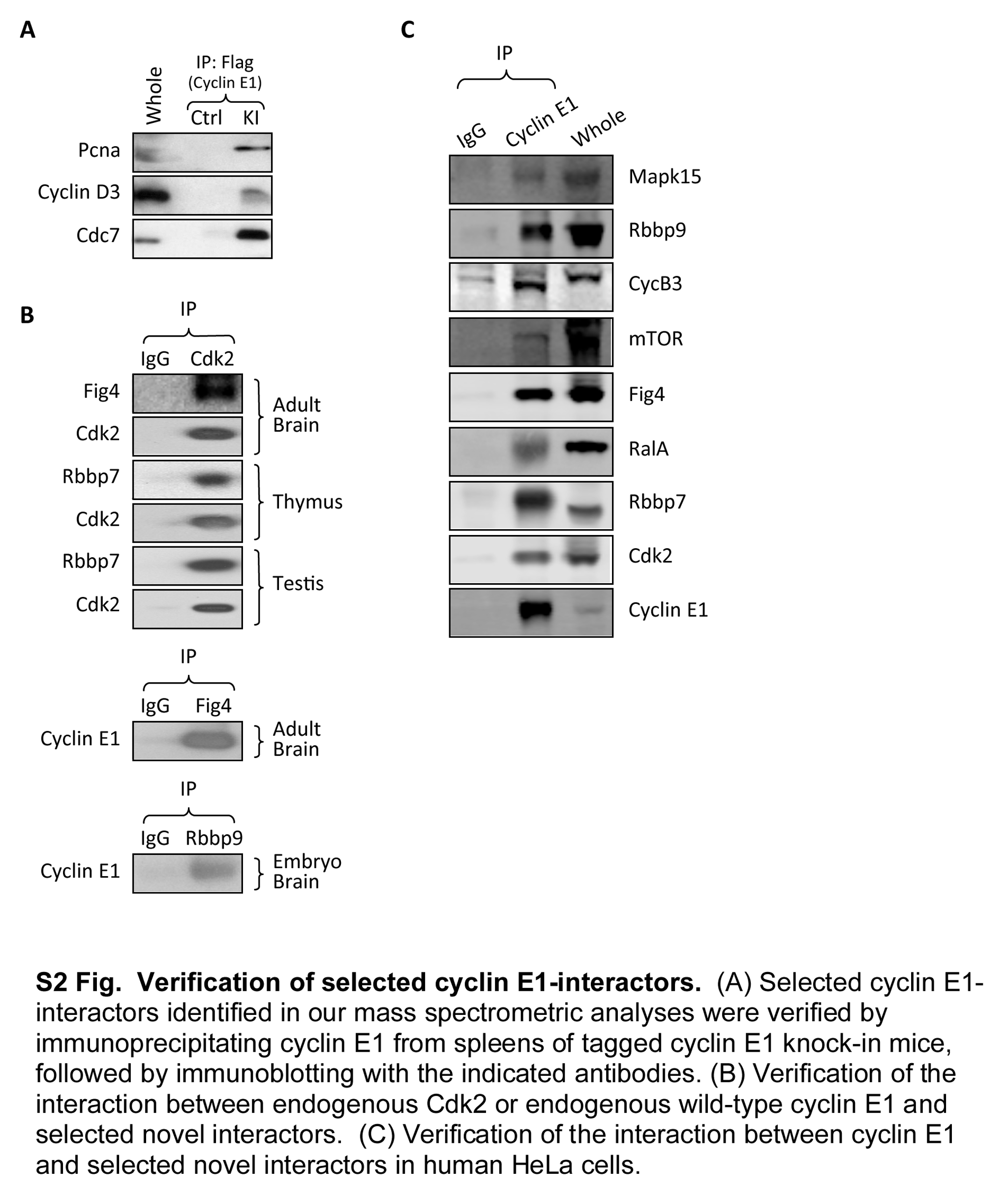

Supplement: S2 Fig — (A) Selected cyclin E1-interactors identified in our mass spectrometric analyses were verified by immunoprecipitating cyclin E1 from spleens of tagged cyclin E1 knock-in mice, followed by immunoblotting with the indicated antibodies. (B) Verification of the interaction between endogenous Cdk2 or endogenous wild-type cyclin E1 and selected novel interactors. (C) Verification of the interaction between cyclin E1 and selected novel interactors in human HeLa cells. (TIF) [file pgen.1006429.s002.tif]

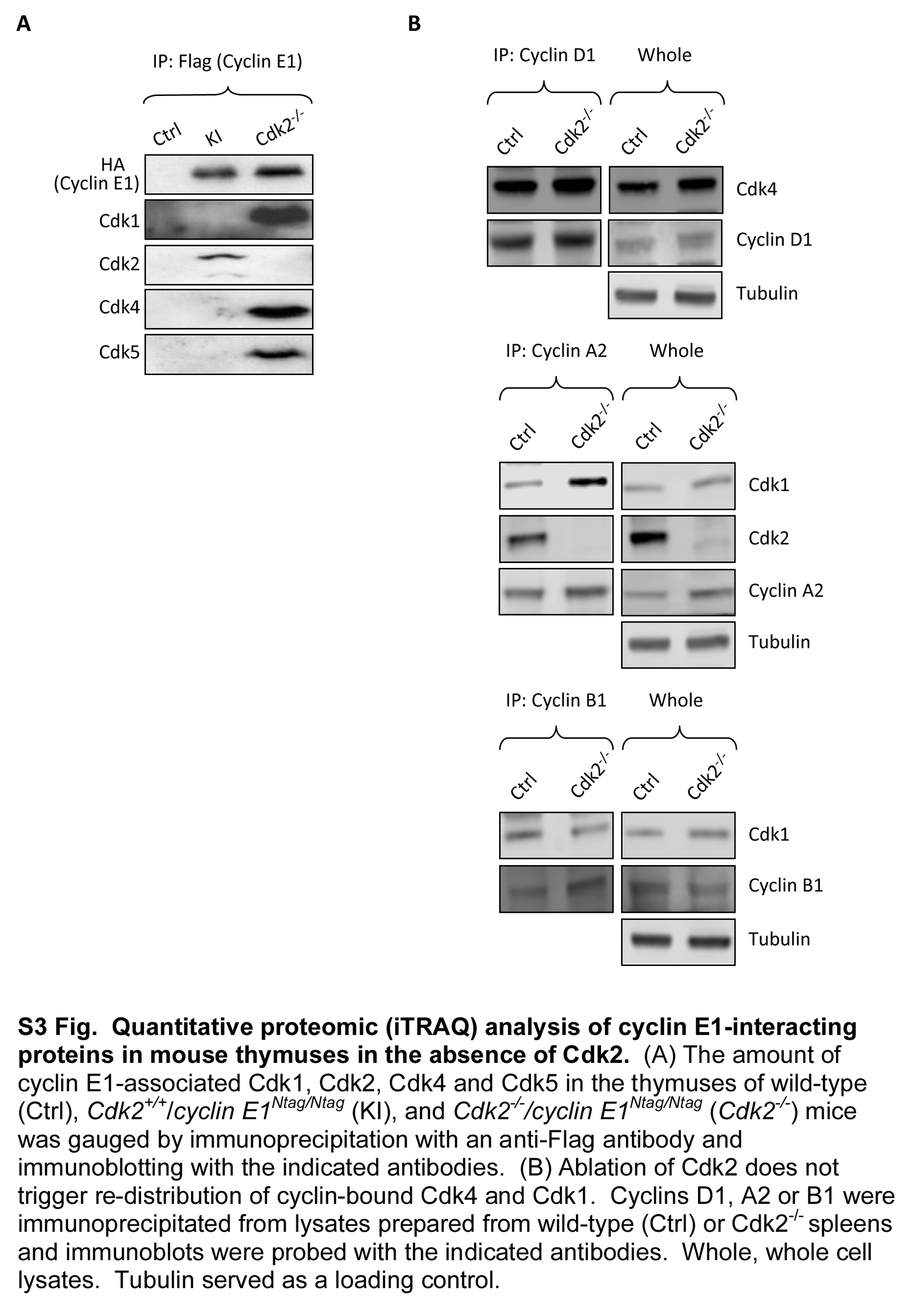

Supplement: S3 Fig — (A) The amount of cyclin E1-associated Cdk1, Cdk2, Cdk4 and Cdk5 in the thymuses of wild-type (Ctrl), Cdk2+/+/cyclin E1Ntag/Ntag (KI), and Cdk2-/-/cyclin E1Ntag/Ntag (Cdk2-/-) mice was gauged by immunoprecipitation with an anti-Flag antibody and immunoblotting with the indicated antibodies. (B) Ablation of Cdk2 does not trigger re-distribution of cyclin-bound Cdk4 and Cdk1. Cyclins D1, A2 or B1 were immunoprecipitated from lysates prepared from wild-type (Ctrl) or Cdk2-/- spleens and immunoblots were probed with the indicated antibodies. Whole, whole cell lysates. Tubulin served as a loading control. (TIF) [file pgen.1006429.s003.tif]

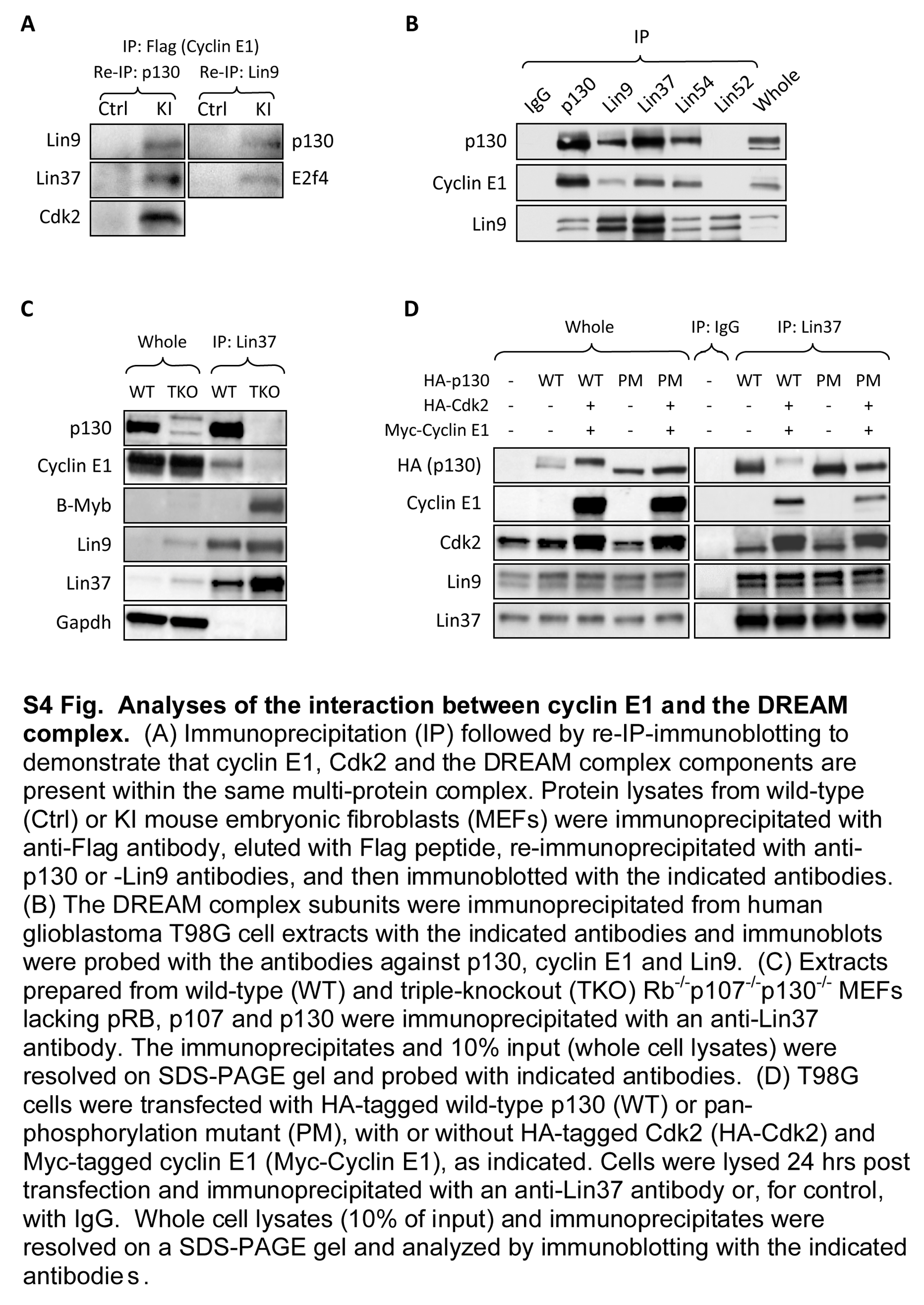

Supplement: S4 Fig — (A) Immunoprecipitation (IP) followed by re-IP-immunoblotting to demonstrate that cyclin E1, Cdk2 and the DREAM complex components are present within the same multi-protein complex. Protein lysates from wild-type (Ctrl) or KI mouse embryonic fibroblasts (MEFs) were immunoprecipitated with anti-Flag antibody, eluted with Flag peptide, re-immunoprecipitated with anti-p130 or -Lin9 antibodies, and then immunoblotted with the indicated antibodies. (B) The DREAM complex subunits were immunoprecipitated from human glioblastoma T98G cell extracts with the indicated antibodies and immunoblots were probed with the antibodies against p130, cyclin E1 and Lin9. (C) Extracts prepared from wild-type (WT) and triple-knockout (TKO) Rb-/-p107-/-p130-/- MEFs lacking pRB, p107 and p130 were immunoprecipitated with an anti-Lin37 antibody. The immunoprecipitates and 10% input (whole cell lysates) were resolved on SDS-PAGE gel and probed with indicated antibodies. (D) T98G cells were transfected with HA-tagged wild-type p130 (WT) or pan-phosphorylation mutant (PM), with or without HA-tagged Cdk2 (HA-Cdk2) and Myc-tagged cyclin E1 (Myc-Cyclin E1), as indicated. Cells were lysed 24 hrs post transfection and immunoprecipitated with an anti-Lin37 antibody or, for control, with IgG. Whole cell lysates (10% of input) and immunoprecipitates were resolved on a SDS-PAGE gel and analyzed by immunoblotting with the indicated antibodies. (TIF) [file pgen.1006429.s004.tif]

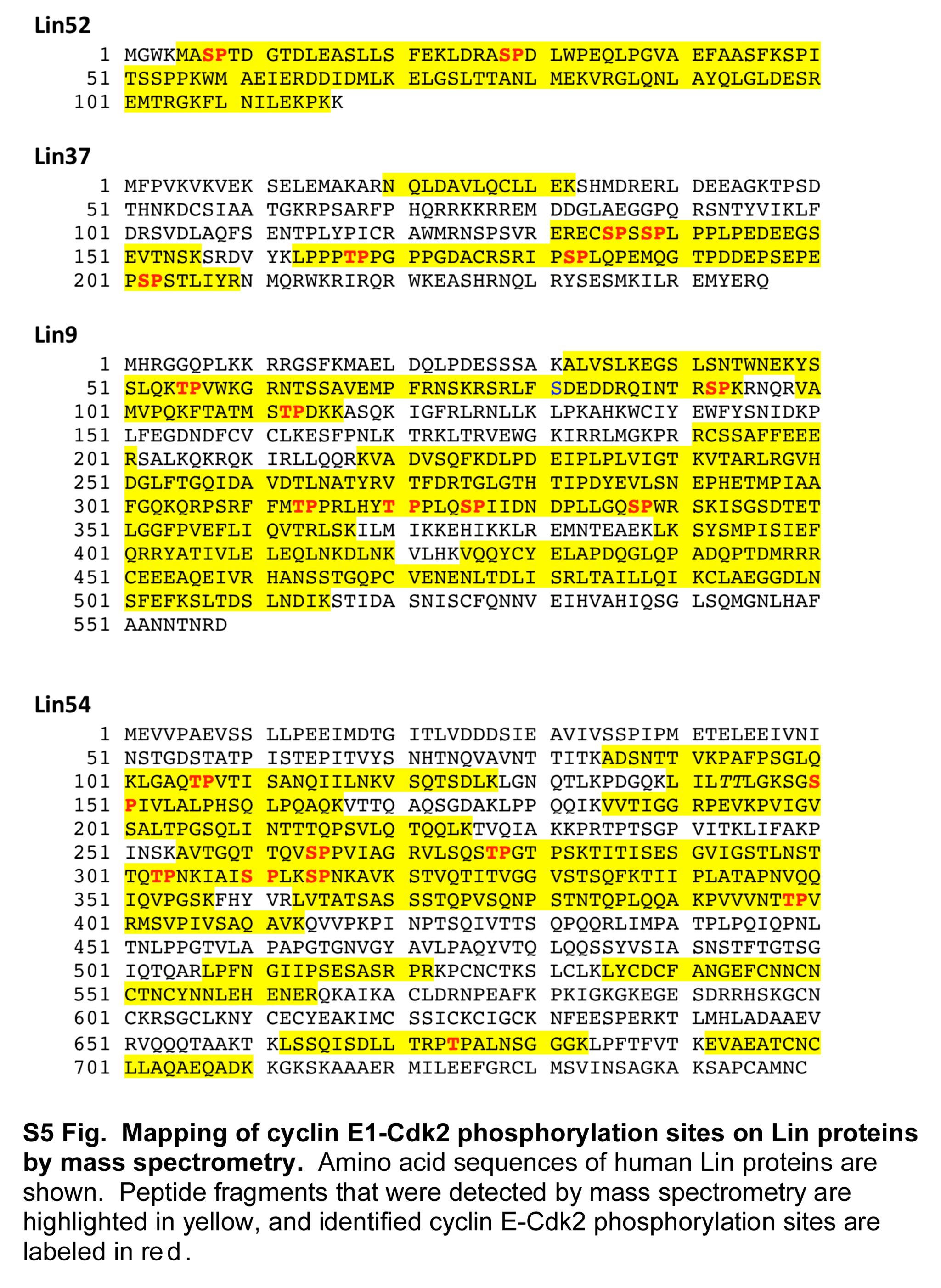

Supplement: S5 Fig — Amino acid sequences of human Lin proteins are shown. Peptide fragments that were detected by mass spectrometry are highlighted in yellow, and identified cyclin E-Cdk2 phosphorylation sites are labeled in red. (TIF) [file pgen.1006429.s005.tif]

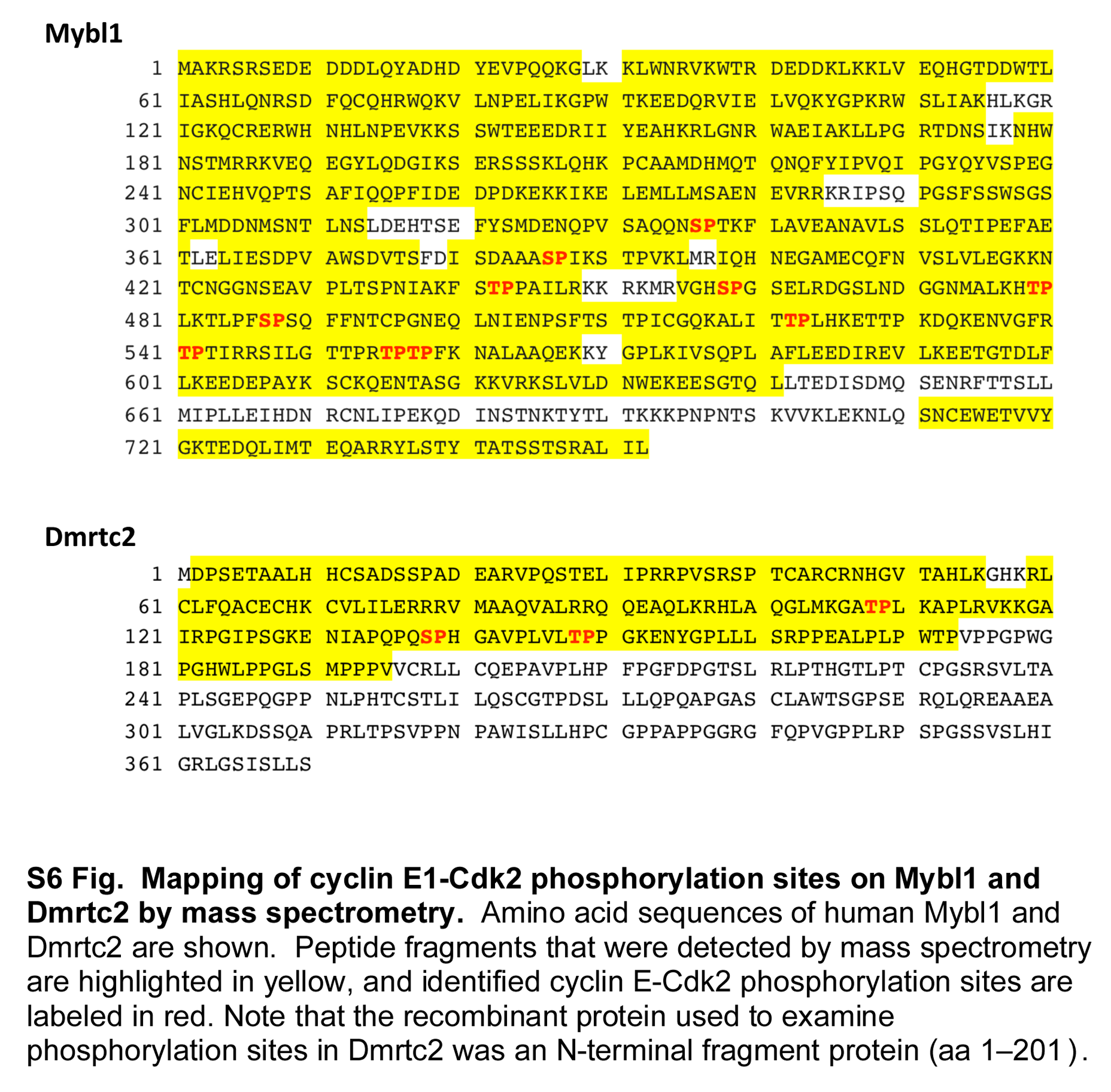

Supplement: S6 Fig — Amino acid sequences of human Mybl1 and Dmrtc2 are shown. Peptide fragments that were detected by mass spectrometry are highlighted in yellow, and identified cyclin E-Cdk2 phosphorylation sites are labeled in red. Note that the recombinant protein used to examine phosphorylation sites in Dmrtc2 was an N-terminal fragment protein (aa 1–201). (TIF) [file pgen.1006429.s006.tif]
