# Supplementary material for: Proteomic Landscape of Tissue-Specific Cyclin E Functions in Vivo
Source: PLoS Genet. 2016 Nov 9;12(11):e1006429. doi: 10.1371/journal.pgen.1006429 (PMC5102403; doi:10.1371/journal.pgen.1006429)
Supplement: S1 Appendix — (DOCX) [file pgen.1006429.s012.docx]

**Supporting Information**

**S1 Fig. Analyses of the five mouse organs used for proteomic analyses.** (A) Sections of adult brains, testes, spleens and thymuses, as well as of embryonic brains, stained for Ki67. (B) Quantification of the fraction of proliferating (Ki67-positive) cells from (A). (C) The levels of Ser 807/811-phosphorylated pRB (Phospho-Rb), total pRB, and cyclin A2 in the five mouse organs. Tubulin served as a loading control.

**S2 Fig. Verification of selected cyclin E1-interactors.** (A) Selected cyclin E1-interactors identified in our mass spectrometric analyses were verified by immunoprecipitating cyclin E1 from spleens of tagged cyclin E1 knock-in mice, followed by immunoblotting with the indicated antibodies. (B) Verification of the interaction between endogenous Cdk2 or endogenous wild-type cyclin E1 and selected novel interactors. (C) Verification of the interaction between cyclin E1 and selected novel interactors in human HeLa cells.

**S3 Fig. Quantitative proteomic (iTRAQ) analysis of cyclin E1-interacting proteins in mouse thymuses in the absence of Cdk2.** (A) The amount of cyclin E1-associated Cdk1, Cdk2, Cdk4 and Cdk5 in the thymuses of wild-type (Ctrl), *Cdk2^+/+^*/*cyclin E1^Ntag/Ntag^* (KI), and *Cdk2^-/-^/cyclin E1^Ntag/Ntag^* (*Cdk2^-/-^*) mice was gauged by immunoprecipitation with an anti-Flag antibody and immunoblotting with the indicated antibodies. (B) Ablation of Cdk2 does not trigger re-distribution of cyclin-bound Cdk4 and Cdk1. Cyclins D1, A2 or B1 were immunoprecipitated from lysates prepared from wild-type (Ctrl) or Cdk2^-/-^ spleens and immunoblots were probed with the indicated antibodies. Whole, whole cell lysates. Tubulin served as a loading control.

**S4 Fig. Analyses of the interaction between cyclin E1 and the DREAM complex.** (A) Immunoprecipitation (IP) followed by re-IP-immunoblotting to demonstrate that cyclin E1, Cdk2 and the DREAM complex components are present within the same multi-protein complex. Protein lysates from wild-type (Ctrl) or KI mouse embryonic fibroblasts (MEFs) were immunoprecipitated with anti-Flag antibody, eluted with Flag peptide, re-immunoprecipitated with anti-p130 or -Lin9 antibodies, and then immunoblotted with the indicated antibodies. (B) The DREAM complex subunits were immunoprecipitated from human glioblastoma T98G cell extracts with the indicated antibodies and immunoblots were probed with the antibodies against p130, cyclin E1 and Lin9. (C) Extracts prepared from wild-type (WT) and triple-knockout (TKO) Rb^-/-^p107^-/-^p130^-/-^ MEFs lacking pRB, p107 and p130 were immunoprecipitated with an anti-Lin37 antibody. The immunoprecipitates and 10% input (whole cell lysates) were resolved on SDS-PAGE gel and probed with indicated antibodies. (D) T98G cells were transfected with HA-tagged wild-type p130 (WT) or pan-phosphorylation mutant (PM), with or without HA-tagged Cdk2 (HA-Cdk2) and Myc-tagged cyclin E1 (Myc-Cyclin E1), as indicated. Cells were lysed 24 hrs post transfection and immunoprecipitated with an anti-Lin37 antibody or, for control, with IgG. Whole cell lysates (10% of input) and immunoprecipitates were resolved on a SDS-PAGE gel and analyzed by immunoblotting with the indicated antibodies.

**S5 Fig.** **Mapping of cyclin E1-Cdk2 phosphorylation sites on Lin proteins by mass spectrometry.**

Amino acid sequences of human Lin proteins are shown. Peptide fragments that were detected by mass spectrometry are highlighted in yellow, and identified cyclin E-Cdk2 phosphorylation sites are labeled in red.

**S6 Fig.** **Mapping of cyclin E1-Cdk2 phosphorylation sites on Mybl1 and Dmrtc2 by mass spectrometry.**

Amino acid sequences of human Mybl1 and Dmrtc2 are shown. Peptide fragments that were detected by mass spectrometry are highlighted in yellow, and identified cyclin E-Cdk2 phosphorylation sites are labeled in red. Note that the recombinant protein used to examine phosphorylation sites in Dmrtc2 was an N-terminal fragment protein (aa 1–201).

**S1 Table. Identification of cyclin E1-associated proteins in various mouse organs by mass spectrometry.**

This table contains six separate worksheets, first five of which show lists of proteins identified in LC-MS/MS analyses from knock-in (KI) or wild-type control (WT) embryonic brains (EB), spleens, testes, thymuses, and adult brains (AB), respectively. Proteins in the ‘core’ (Category 1) group are highlighted in green, and those in Categories 2 and 3 in yellow and blue, respectively. Each row lists protein name, gene name, STRING name (mouse and human), gene ID, the number of peptides found in KI (Peptides KI) and WT (Peptides WT), the number of detections across experiments for KI (KI detected) and for WT (WT detected), p-value tissue, ratio tissue, p-value all, and ratio all (see S1 Appendix for details of statistical tests). The penultimate column identifies proteins (marked “Y”) that were included into the ‘core’ Category 1 group based on their identification as a highest-confidence interactor in another organ (see S1 Appendix). The last column identifies proteins from Categories 2 and 3 (marked “Y”) that were included into the interactome based on known, STRING-verified interaction with at least one of the ‘core’ interactors. The last worksheet lists 117 proteins from the combined cyclin E1 interactome in all organs.

**S2 Table. Analyses of cyclin E1-interactors.**

This table contains three separate worksheets. The first shows the list of proteins present in the cyclin E1 interactome in all organs. For each cyclin E1- interactor, gene name is listed in the first column, and the major Gene Ontology term assigned to generate Fig 3D in the second column. The third column identifies proteins that were predicted as high- or medium-stringency putative Cdk phosphorylation substrates predicted by Scansite 3.0 (marked ‘High’ and ‘Medium’, respectively). Also shown are organs in which a given protein was identified as cyclin E1-interactor. EB, embryonic brain; AB, adult brain. The second (High) and third (Medium) worksheets list Gene Ontology terms enriched among high stringency and medium stringency putative Cdk phosphorylation substrates, respectively.

**S3 Table. Biological process/molecular function enrichment heat map of cyclin E1 interactors.**

This table contains six separate worksheets and lists Gene Ontology terms enriched among cyclin E1-interactors detected in the indicated organs. EB, embryonic brain; AB, adult brain.

**S4 Table. iTRAQ quantitative comparison of spleen samples.**

Each row corresponds to a different protein. Shown are: (A) Accession number; (B) Protein name; (C) Ratio of the relative abundance of a given protein between *cyclin E1^Ntag/Ntag^* purification products (KI) versus mock purification (WT); (D) Ratio of the relative abundance of a given protein between *Cdk2*^-/-^/*cyclin E1^Ntag/Ntag^* purification products (Cdk2KO) versus *cyclin E1^Ntag/Ntag^* purification products (KI); (E) For each protein, Cdk2KO:KI ratio was normalized against the abundance of cyclin E1 in Cdk2KO and KI purification products.

**S5 Table. Primers used for RT-qPCR.**

The table lists the forward and reverse primers used for RT-qPCR (Fig 7E and 7F).

**Supplemental Experimental Procedures**

**Proteomic data analyses**

**Definition of the number of peptides.** For each protein identified in LC-MS/MS, we defined the number of peptides as the total number of different peptides detected in a single LC-MS/MS experiment (run). For our statistical tests described below, we considered two sets of control data. The first set, *control organ*, was formed by all mass spectrometry runs obtained in the same control organ (6 to 11 independent runs). The second set, *control all*, was formed by combining all mass spectrometry runs for all control organs, which consisted of a total of 39 runs.

**Construction of the cyclin E1 interactome.** We performed the following tests and determined the following parameters: (1) Mann-Whitney U-test to compare in each organ and for each protein the number of peptides in knock-in versus in control organs (*p-value organ*); (2) Mann-Whitney U-test to compare in each organ and for each protein the number of peptides in knock-in versus *control all (p-value all*); (3) *# peptides*, maximum number of peptides detected in the knock-in data for a given protein, in a given organ, in any experiment; (4) *Ratio organ*, for each protein and organ, the ratio of the average number of peptides in knock-in samples across experiments and the sum of the same averages in knock-in and control organ; (5) *Ratio all*, for each protein and organ, the ratio of the average number of peptides in knock-in samples across experiments and the sum of the same averages in knock-in for the same organ and *control all*.

Proteins fulfilling the one of the following two criteria were selected as ‘core’ Category 1, high-confidence interactors: Criterion #1, p < 0.05 for *p-value organ*, and p < 0.05 for *p-value all*, and ≥ 2 peptides in *# peptides*, and ratio > 0.8 in *ratio organ,* and ratio > 0.8 in *ratio all*; OR Criterion #2, p < 0.05 for *p-value all*, and ≥ 1 peptides in *# peptides*, and ratio > 0.8 in *ratio organ,* and ratio > 0.8 in *ratio all,* and Criterion #1 fulfilled in another organ.

As lower confidence, Category 2 interactors we considered proteins that met the following less stringent criteria: p < 0.05 for *p-value organ*, and p < 0.05 for *p-value all*, and 1 peptide in *# peptides*, and ratio > 0.8 in *ratio organ,* and ratio > 0.8 in *ratio all,* and which did not fulfill Criterion #1 in any organ.

As Category 3 interactors we considered proteins that fulfilled the following conditions: p < 0.05 for *p-value all*, and ≥ 1 peptides in *# peptides*, and ratio > 0.8 in *ratio organ,* and ratio > 0.8 in *ratio all,* and which did not fulfill Criterion #1 in any organ.

For each organ, all proteins from Categories 1, 2 and 3 were analyzed using both mouse and human STRING databases (<http://string-db.org/>). We searched for STRING-verified interactions between members of high-confidence ‘core’ Category 1 and proteins in Categories 2 and 3. We only considered interactions that had experimental evidence, with a threshold of 0.15. Any proteins from Categories 2 and 3 that were shown to interact with ‘core’ Category 1 proteins in the STRING database using these criteria were included into the interactome (along with all Category 1 proteins). Such generated organ-specific interactomes are shown in Fig 2A. We also merged all five organ-specific interactomes and constructed an integrated cyclin E1 interactome containing a total of 117 interactors, as shown in Fig 2B.

**Identification of phosphosites by mass spectrometry**

After subjecting GST-Lin9, GST-Lin37, GST-Lin52, GST-Lin54, GST-Mybl1 and GST-Dmrtc2 to *in vitro* kinase reaction with cyclin E1-Cdk2 in the presence of ATP, protein samples were digested with trypsin or thermolysin, and phosphorylation sites were identified by mass spectrometry.

**Cdk2 substrate enrichment analyses**

To determine the fractions of known Cdk2 substrates among cyclin E1-interactors and in the whole proteome, we intersected the list of cyclin E1-interactors with the list of known Cdk2 substrates (Kinase_Substrate_Dataset.gz) obtained from PhosphoSitePlus (Cell Signaling Technology). For the whole proteome, we assumed the total number of proteins to be 20,000 (19,999 excluding cyclin E1). Fractions of Cdk2 substrates among cyclin E1 interactors and in the whole proteome were then compared using one-tailed Fisher’s exact test.

To compare the fraction of predicted Cdk substrates among cyclin E1-interactors versus in the whole proteome, we screened amino acid sequences of cyclin E1-interactors using Scansite 3.0 under high or medium stringency scoring. Selected Cdk phosphorylation motifs are [ST]Px[KR]x (Cdk1 motif 1) and [ST]PxxK (Cdk1 motif 2). Median scores for high and medium stringency searches were 0.252 and 0.341, respectively. For the entire proteome, we searched a Scansite sequence database (SwissProt) for mouse proteins containing at least one of the above two motifs. The resulting predicted Cdk substrates had a median score of 0.536, indicating even less stringent criterion than the above medium-stringency search. P-value was calculated using one-tailed Fisher’s exact test, based on the probability of finding possible Cdk2 substrates in the cyclin E1 interactome.

**Gene Ontology enrichment**

Gene Ontology (GO) enrichment analysis was performed using the DAVID web server (<https://david.ncifcrf.gov/>). For each organ, we used the list of proteins selected to generate cyclin E1 interactome (see above, Construction of the Cyclin E1 Interactome). We used default settings in DAVID to select statistically significant categories (EASE score, a modified Fisher’s exact test, < 0.1).

**T98G synchronization experiment**

Cell synchronization and release experiment was performed as described in [1]. Briefly, exponentially growing human glioblastoma T98G cells were plated in 150 mm dishes (3 x 10^6^ cells per dish) and allowed to grow for 36 hrs until they were 60% confluent. Cells were then incubated in serum-free medium (0% FBS) for 72 hrs, after which the control dishes of serum starved cells were harvested. The remaining dishes were released from growth arrest by adding 20% FBS supplemented either with 0.2% DMSO (vehicle control) or 20 µM CVT-313 inhibitor, and harvested after 6, 10, 14, 18 and 24 hrs. Cells were lysed in EBC buffer (50 mM Tris-HCl pH 8.0, 150 mM NaCl, 0.5% NP-40, 0.5 mM EDTA and 0.01% 2-mercaptoethanol) [1] supplemented with protease and phosphatase inhibitor cocktails, and immunoprecipitated with an anti-Lin37 antibody or with control rabbit IgG (1 μg/ml). Whole cell lysates (inputs) and immunoprecipitates were resolved on 4-15% gradient Criterion gels (BioRad), transferred to nitrocellulose membranes and probed with the indicated antibodies.

**T98G cell transfection**

T98G cells were transfected in 100 mm dishes at 80% confluency using PEI transfection reagent (Polysciences, Inc) and 6 µg of total DNA including HA-p130-WT-pcDNA3.0 or HA-p130-PM19A-pcDNA3.0 (2µg) [gift form Dr. J. Bartek [2]), HA-Cdk2-WT-pCMV (2µg) and 6Myc-Cyclin E1-pcDNA3.0 (2µg), or 4µg of GFP-pcDNA3.0 control vector as a filler plasmid. 24 hrs post transfection, cells were harvested in EBC buffer, immunoprecipitated using an anti-Lin37 antibody or with control rabbit IgG, resolved on 4-15% gradient SDS-PAGE gels followed by immunoblotting with anti-HA, cyclin E1, Cdk2, Lin9 and Lin37 antibodies.

**Quantification of Ki67-positive cells**

Formalin-fixed organs were paraffin-embedded, sectioned and stained with Ki67 antibody at the Specialized Histopathology Core (Dana-Farber/Harvard Cancer Center, Boston, MA). For quantification, images were randomly taken at 100x magnification. Ki67-positive and -negative cells were manually counted from about 10 images for each organ (>1,000 cells).

**Supplemental References**

1. Litovchick L, Sadasivam S, Florens L, Zhu X, Swanson SK, et al. (2007) Evolutionarily conserved multisubunit RBL2/p130 and E2F4 protein complex represses human cell cycle-dependent genes in quiescence. Mol Cell 26: 539-551.

2. Hansen K, Farkas T, Lukas J, Holm K, Ronnstrand L, et al. (2001) Phosphorylation-dependent and -independent functions of p130 cooperate to evoke a sustained G1 block. Embo J 20: 422-432.
